# Synthesis and circularly polarized luminescence properties of BINOL-derived bisbenzofuro[2,3-*b*:3’,2’-*e*]pyridines (BBZFPys)

**DOI:** 10.3762/bjoc.16.32

**Published:** 2020-03-06

**Authors:** Ryo Takishima, Yuji Nishii, Tomoaki Hinoue, Yoshitane Imai, Masahiro Miura

**Affiliations:** 1 Department of Applied Chemistry, Graduate School of Engineering, Osaka University, Suita, Osaka 565-0871, Japan; 2 Frontier Research Base for Global Young Researchers, Graduate School of Engineering, Osaka University, Suita, Osaka 565-0871, Japan; 3 Department of Applied Chemistry, Faculty of Science and Engineering, Kindai University, Higashi-Osaka, Osaka 577-8502, Japan

**Keywords:** BINOL, C–H activation, circularly polarized luminescence, palladium

## Abstract

A series of optically active bisbenzofuro[2,3-*b*:3’,2’-*e*]pyridine (BBZFPy) derivatives was synthesized starting with the readily available (*S*)- and (*R*)-1,1’-bi-2-naphthols through a palladium-catalyzed multiple intramolecular C–H/C–H coupling as the key ring-closure step. The effect of terminal *tert*-butyl substituents on the BBZFPy skeleton was systematically investigated to uncover a unique aggregation-induced enhancement of CPL characteristics in the solid state. The crystal structures of the coupling products were also evaluated by single crystal X-ray analysis and the well-ordered intermolecular stacking arrangements appeared to be responsible for the enhanced CPL.

## Introduction

**Scheme 1:**
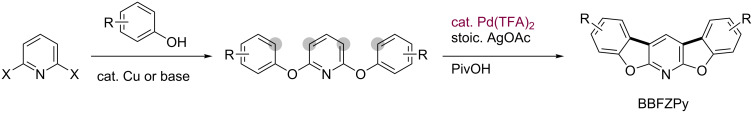
Synthesis of BBFZPys through the Pd-catalyzed C–H/C–H coupling.

Densely-fused (hetero)aromatic compounds have been a key motif in a wide range of manufactured functional molecules, as they exhibit fundamentally useful electrochemical and photophysical properties. Considerable effort has therefore taken into the development of efficient methods for the construction of such polycyclic scaffolds, and the last decade has witnessed a remarkable improvement in the palladium-catalyzed C–H/C–H oxidative coupling as one of the potential synthetic strategies [1]. This method is straightforward and highly step-economical, enabling us to produce condensed (hetero)acenes from rather simple polyarenes, in which several aromatic units are connected with each other through appropriate linker units [2–11]. Recently, we reported the synthesis and optical properties of a series of furan-fused aromatics via the formal dehydrogenative coupling adopting oxygen atom as the linker [12–17]. In particular, bisbenzofuro[2,3-*b*:3′,2′-*e*]pyridines (BBZFPys) were found to exhibit intense photoluminescence with relatively high quantum efficiency (Φ_flu_ up to 0.70), indicating that the BBFZPy scaffold may serve as a key fluorophore unit in certain light-emitting functional materials (Scheme 1) [14].

Meanwhile, organic optoelectronic materials with circularly polarized luminescence (CPL) characteristics have attracted significant research interests in recent years [18–21] for their potential applications in three-dimensional displays [22], information storage systems [23], molecular photoswitches [24], etc. Among a series of chiral scaffolds for CPL emitting molecules, axially chiral 1,1’-bi-2-naphthol (BINOL) has been frequently adopted for the core structure owing to the availability of both enantiomers as well as the ease of site-selective functionalization. Up to date, many BINOL-based CPL active compounds have been established by installing aromatic subunits on the periphery of the binaphthyl skeleton or on the hydroxy groups [25–32], extending the π-system [33], and linearly connecting the naphthyl rings [34–36]. In these compounds, the hydroxy groups are remained untouched or protected as the corresponding ethers or esters. We envisioned that the assembly of the binaphthyl-fused furan motif embedding the BINOL hydroxy groups into the polyaromatic scaffolds would lead to the development of new chiroptical materials. Such molecules, however, have hardly been investigated to date probably because of the synthetic difficulty to obtain them as pure enantiomers. There have been only a few reports for the binaphthyl-fused furan-ring construction from the C3-alkynylated BINOL derivatives [37–39]. In this context, we herein describe the synthesis of axially chiral BINOL-derived BBZFPys through the palladium-catalyzed oxidative coupling reaction. The optical properties of the synthesized polyaromatic compounds were systematically studied, and some of them displayed an interesting aggregation-induced enhancement of CPL in the solid state with considerably higher dissymmetry factors compared to those in solution.

## Results and Discussion

### Synthesis of BINOL-derived BBFPys

**Scheme 2:**
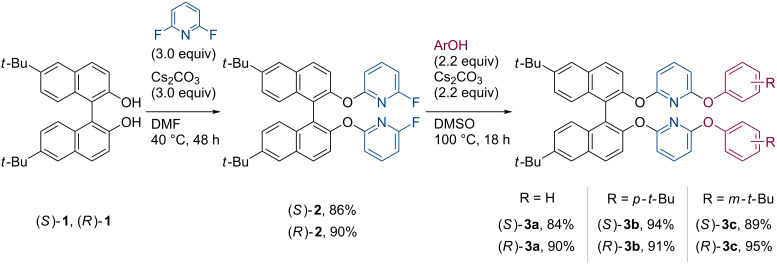
Synthesis of **3a**–**c**.

**Scheme 3:**
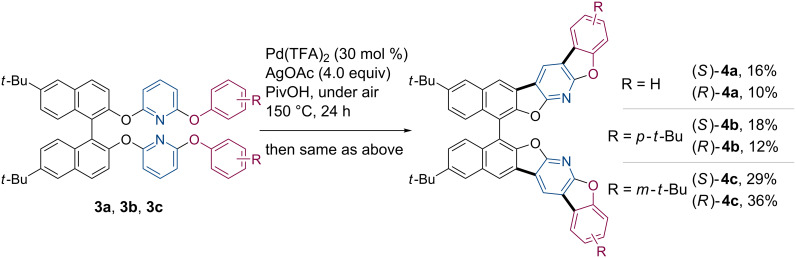
Synthesis of **4a**–**c** through oxidative coupling reaction.

The study was initiated with the synthesis of 2,6-diaryloxypyridines **3** bearing a 1,1’-binaphthyl backbone as precursors for the dehydrogenative coupling reaction (Scheme 2). In general, functionalization of the BINOL hydroxy groups should be performed at temperatures below 80 °C to prevent racemization [40–41]. 6,6’-Di-*tert*-butyl-1,1’-bi-2-naphthol (**1**) was treated with 2,6-difluoropyridine using cesium carbonate as base in DMF at 40 °C [42], giving both the enantiomers of **2** in optically pure forms. The remaining fluorine substituents were subsequently replaced by a series of phenols including unsubstituted phenol, *p*-*tert*-butylphenol, and *m*-*tert*-butylphenol to produce the corresponding unsymmetrically substituted pyridines **3a**–**c** in high yields. We then examined the oxidative cyclization of these compounds under the standard conditions adopting Pd(TFA)_2_ (30 mol %, TFA = trifluoroacetate) and AgOAc (3.0 equiv) as catalyst and oxidant, respectively, in pivalic acid as solvent (Scheme 3). Since the desired four-fold coupling products **4** were obtained only in small quantities after the reactions, the crude mixtures containing incompletely cyclized compounds were again subjected to the same catalytic conditions. To our delight, all the target molecules **4a**–**c** were successfully isolated as pure enantiomers in 10–36% yields. The higher yield of **4c** was probably due to its better solubility.

**Scheme 4:**
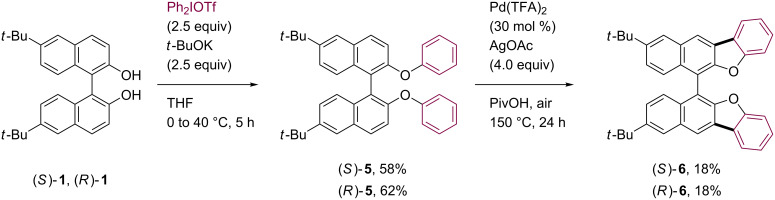
Synthesis of **6**.

In order to systematically evaluate the optical properties of these coupling products, a simple benzofuran-fused 1,1’-binaphthyl **6** was also synthesized as a benchmark (Scheme 4). The parent ether **5** was obtained through the arylation of **1** utilizing Ph_2_IOTf as arylating reagent [43–44]. Some copper-mediated arylation protocols using bromobenzene or iodobenzene [45–46] were also applicable to the preparation of **5**, but significant loss of optical purity was inevitable. After the Pd-catalyzed cyclization under the standard conditions, the desired compounds (*S*)- and (*R*)-**6** were obtained as pure enantiomers in 18% yield.

### Optical properties

**Figure 1:**
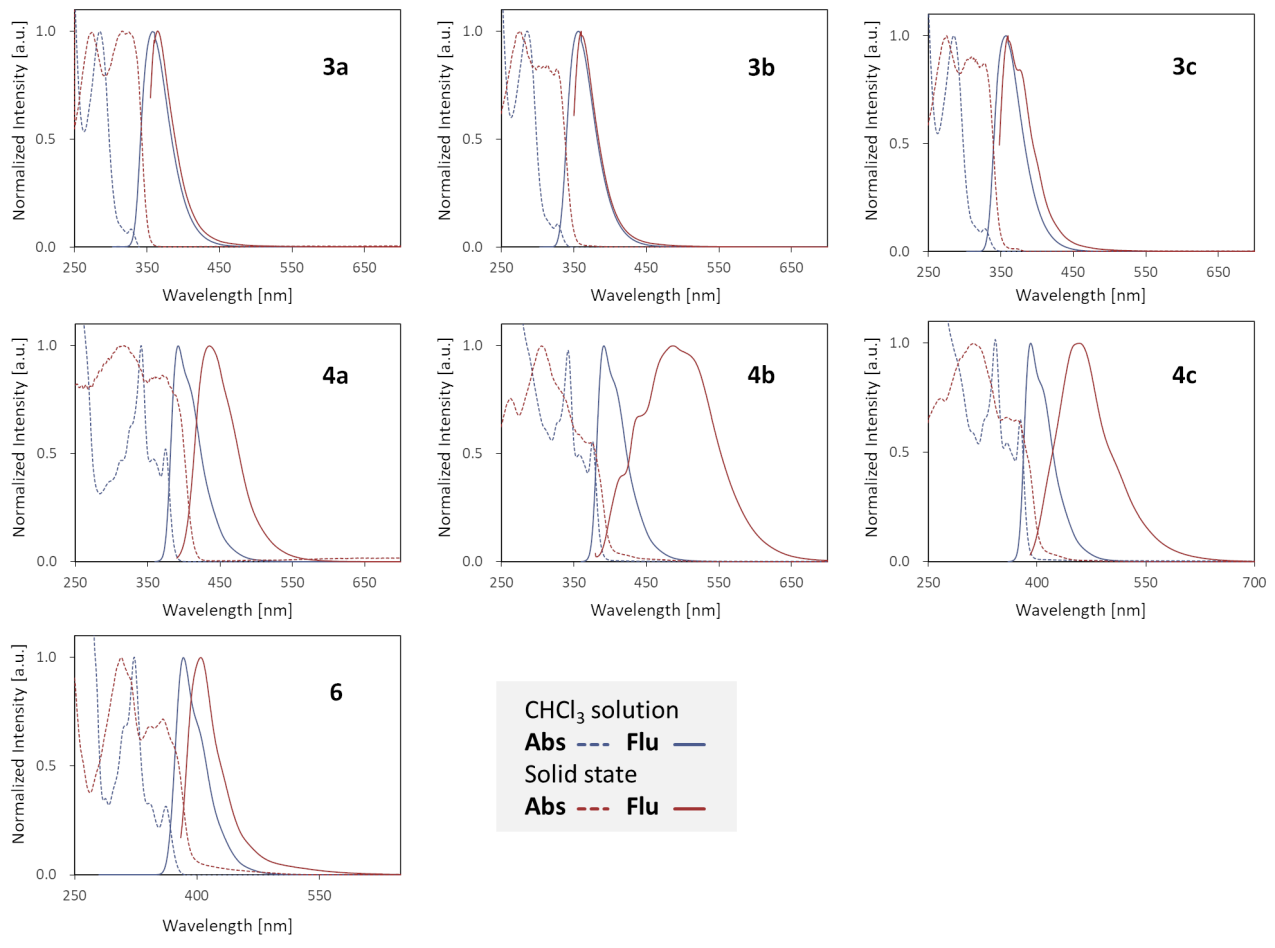
Absorption (dotted line) and fluorescence (solid line) spectra of **3**, **4**, and **6** measured as CHCl_3_ solutions (1.0 × 10^−5^ M) and in solid states.

We next investigated the optical properties of the coupling products (Figure 1 and Table 1). The parent compounds **3a**–**c** emitted fluorescence at around 360 nm both in sufficiently diluted CHCl_3_ solutions (1.0 × 10^−5^ M) and in the solid states. The quantum yields of these molecules were around 0.15 in solution, which is typical for binaphthyl compounds [47]. In contrast, **4a**–**c** as well as **6** exhibited fluorescence at around 390 nm in solution, with relatively higher quantum yields of 0.37–0.40. The emission bands of **4a**–**c** in their solid state were considerably red-shifted as compared to that of **6**, suggesting that these compounds displayed the appreciable effect of molecular aggregation. Interestingly, **4b** and **4c**, bearing the additional terminal *tert*-butyl substituents, were more red-shifted than **4a** despite such a sterically demanding group usually disturbs intermolecular stacking interactions.

**Table 1:** Florescence properties.^a^

Compd.	solution λ_max_ (λ_ex_)	solid λ_max_ (λ_ex_)	Φ (solution)	Φ (solid)

**3a**	358 nm (282 nm)	360 nm (341 nm)	0.13	0.30

**3b**	357 nm (283 nm)	360 nm (338 nm)	0.14	0.21

**3c**	357 nm (283 nm)	360 nm (338 nm)	0.16	0.19

**4a**	392 nm (341 nm)	436 nm (369 nm)	0.39	0.17

**4b**	391 nm (342 nm)	488 nm (369 nm)	0.38	0.08

**4c**	391 nm (342 nm)	457 nm (370 nm)	0.40	0.07

**6**	384 nm (263 nm)	405 nm (380 nm)	0.37	0.13

^a^Measured at room temperature as solution in CHCl_3_ (1.0 × 10^−5^ M) and in the solid states.

**Figure 2:**
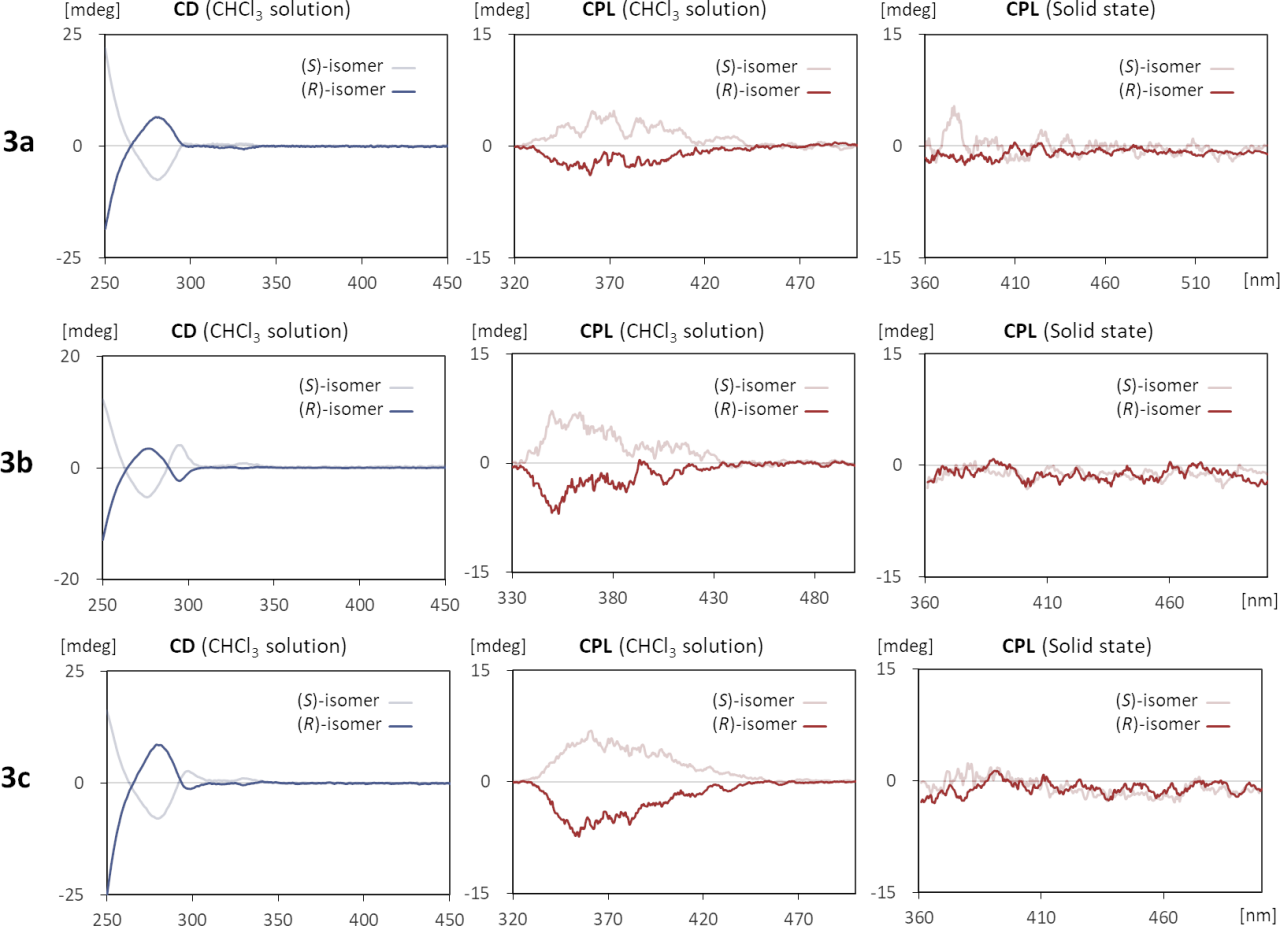
CD and CPL spectra of **3** measured as CHCl_3_ solutions (1.0 × 10^−5^ M) and in the solid states (dispersed in Fomblin^®^).

**Figure 3:**
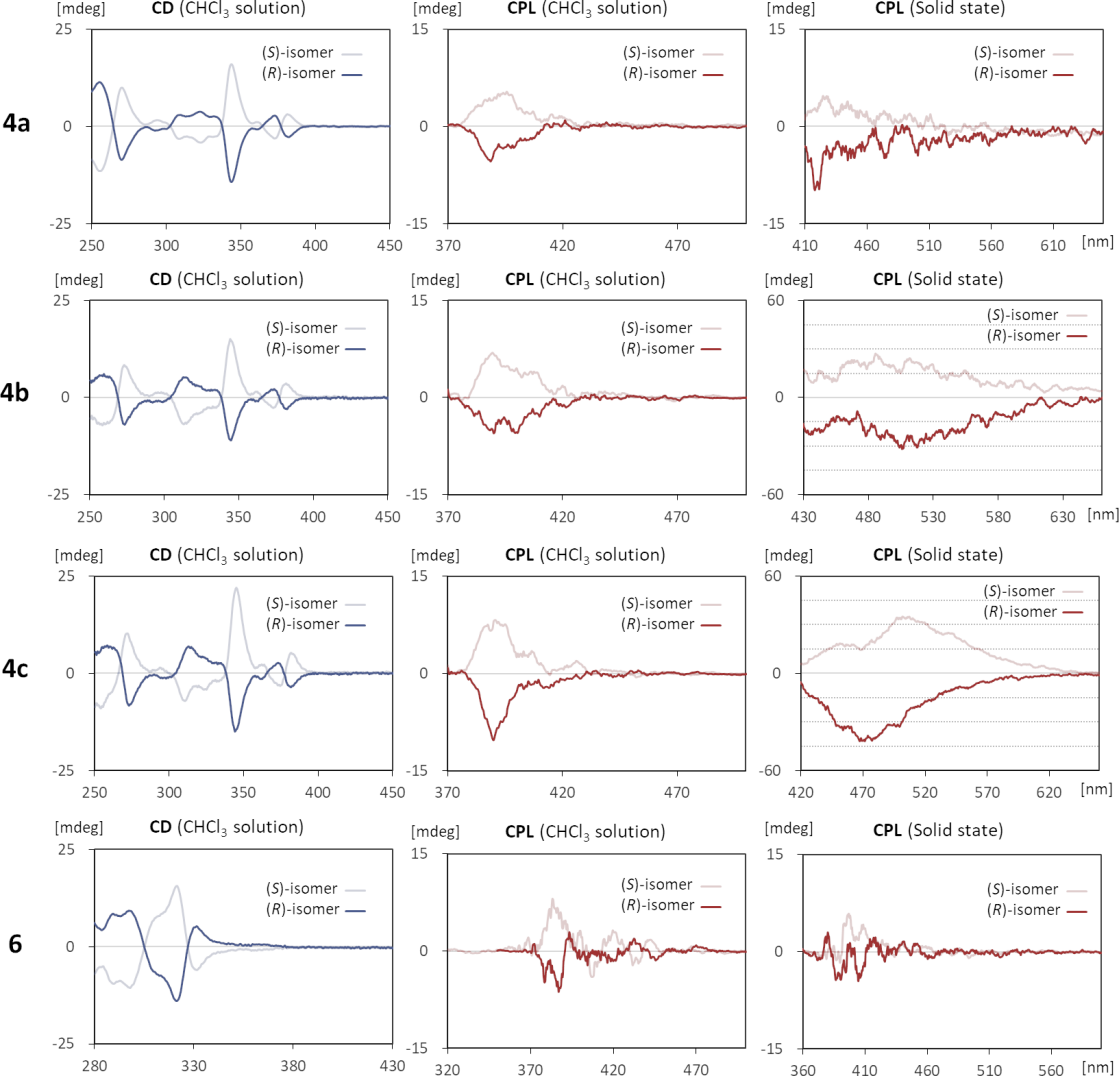
CD and CPL spectra of **4** and **6** measured as CHCl_3_ solutions (1.0 × 10^−5^ M) and in solid states (dispersed in Fomblin^®^).

Subsequently, the chiroptical properties of the synthesized compounds were evaluated (Figure 2 and Figure 3, Table 2). The circular dichroism (CD) spectra in CHCl_3_ solutions showed apparent Cotton signals characteristic to axially chiral molecules. In all cases, the (*S*)- and (*R*)-enantiomers were evidently mirror images of each other while the anisotropy factors *g*_abs_ are relatively small and in the range of 10^−4^ to 10^−7^. The spectral shapes of **3a**–**c** and **4a**–**c** were respectively comparable, indicating that the posted positions of the terminal *tert*-butyl groups exerted minimal influence on the Cotton effect in the solution state. A similar trend was observed for the CPL spectra. The (*S*)-isomers displayed left-handed CPL characteristics throughout the wavelengths of their corresponding fluorescence emission bands, whereas the (*R*)-isomers emitted right-handed CPL to produce the mirror images. The calculated luminescence dissymmetry factors [48] *g*_lum_ for the solutions were all within the range of 3.80 × 10^−4^ to 6.90 × 10^−4^. On the other hand, in the dispersed solid state in Fomblin^®^ PFPE (perfluoropolyether) fluid (each sample was not soluble in the fluid and gave the expected solid-state luminescence), the signal intensity drastically changed depending on the molecular structures. In particular, **4b** and **4c** exhibited enhanced CPL characteristics with considerably high *g*_lum_ values of 6.68 × 10^−3^ and 6.06 × 10^−3^, respectively, which were approximately ten times larger than those of the CHCl_3_ solutions [49]. The parent compounds **3a**–**c**, however, did not give clear mirror images in the CPL measurements. Since such a phenomenon was not observed for **4a** and **6**, the terminal *tert*-butyl substituents in **4b** and **4c** were likely to assist the formation of well-ordered aggregates, being consistent with the observation of the red-shifted luminescence discussed above.

**Table 2:** Calculated dimensionless dissymmetry factors.^a^

Compd.	*g*_abs_ (solution)	*g*_lum_ (solution)	*g*_lum_ (solid)

**3a**	8.72 × 10^−7^ (285 nm)	4.37 × 10^−4^ (358 nm)	n.d.^b^

**3b**	3.18 × 10^−7^ (285 nm)	6.72 × 10^−4^ (357 nm)	n.d.^b^

**3c**	1.06 × 10^−6^ (285 nm)	6.90 × 10^−4^ (357 nm)	n.d.^b^

**4a**	5.60 × 10^−6^ (341 nm)	5.57 × 10^−4^ (392 nm)	5.40 × 10^−4^ (436 nm)

**4b**	9.96 × 10^−5^ (342 nm)	4.60 × 10^−4^ (391 nm)	6.68 × 10^−3^ (488 nm)

**4c**	8.72 × 10^−5^ (342 nm)	6.40 × 10^−4^ (391 nm)	6.06 × 10^−3^ (457 nm)

**6**	1.00 × 10^−4^ (256 nm)	3.80 × 10^−4^ (384 nm)	1.40 × 10^−4^ (405 nm)

^a^Measured at room temperature as solution in CHCl_3_ (1.0 × 10^−5^ M) and in solid states (dispersed in Fomblin^®^). ^b^Not determined.

### Crystal structures of **4b** and **4c**

**Figure 4:**
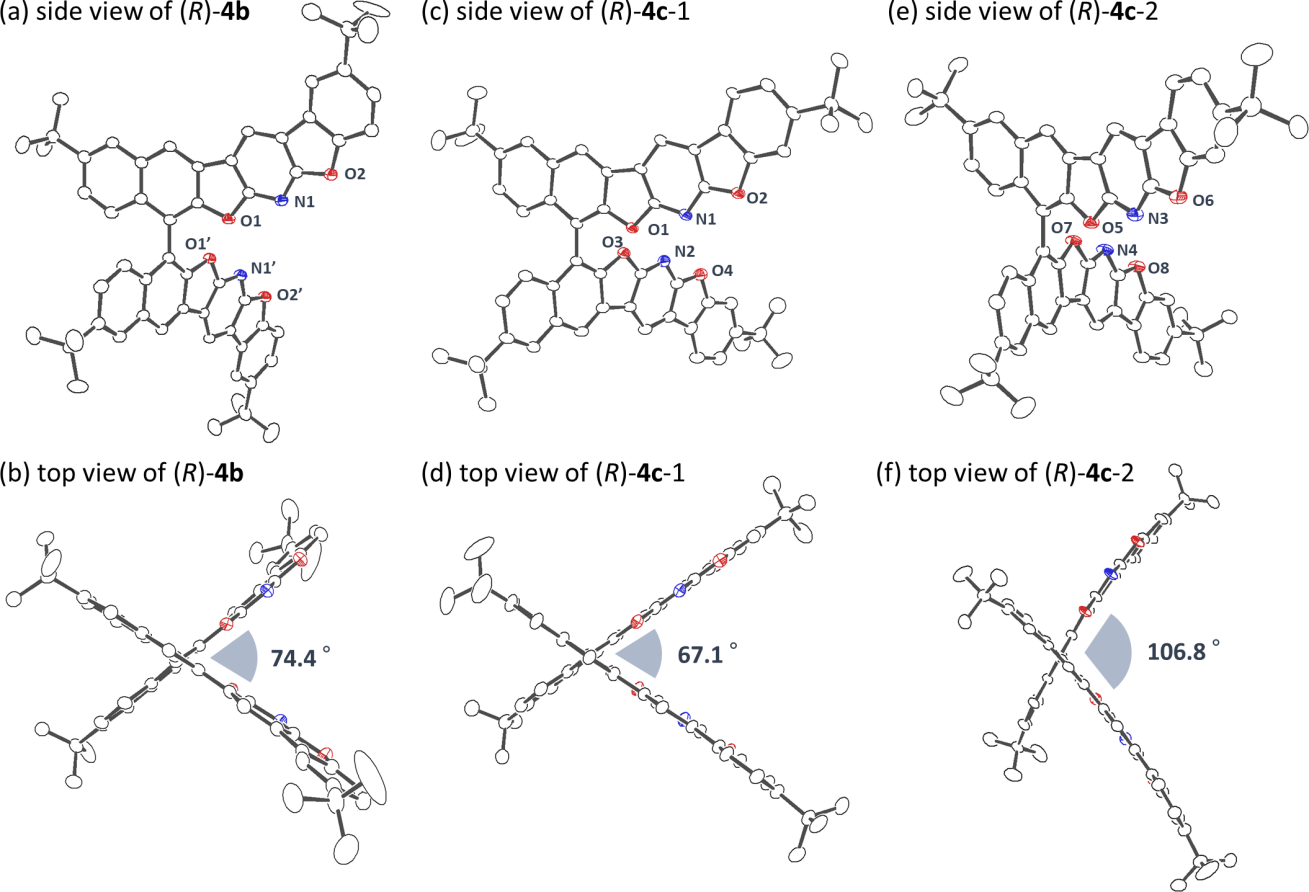
ORTEP drawings of **4b** and **4c** with 50% thermal probability. Hydrogen atoms and solvent molecules are omitted for clarity. Only major orientation of the disordered structure is displayed. The CCDC numbers are 1971471 for (*R*)-**4b** and 1971470 for (*R*)-**4c**.

**Figure 5:**
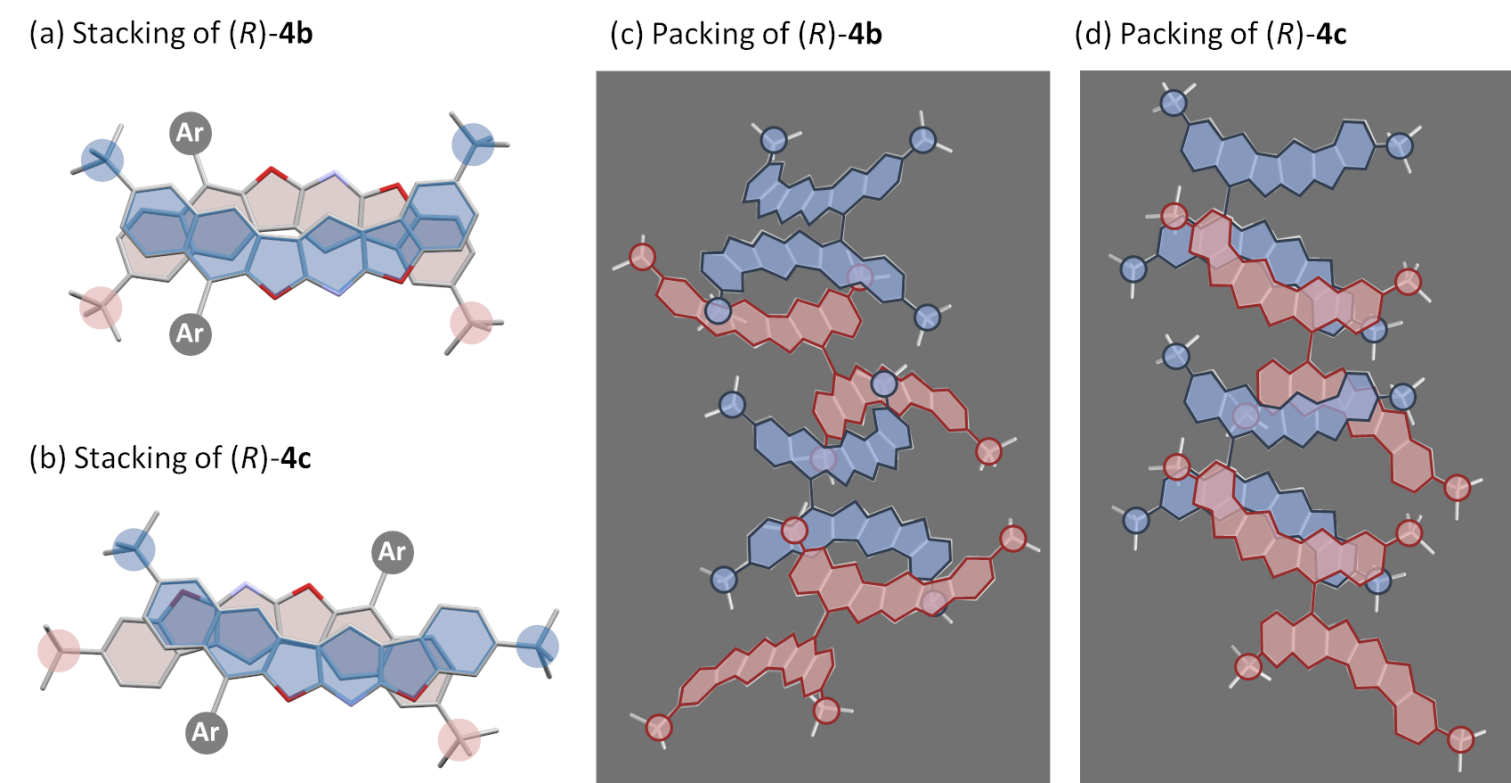
Intramolecular stacking structures of **4b** and **4c**.

The molecular structures of **4b** and **4c** were unambiguously determined by single crystal X-ray diffraction analysis. The crystal of **4b** is classified into a space group *P*4_3_22 (tetragonal) with a biaryl torsion angle of 74.4° (Figure 4b). A considerable intermolecular π–π stacking interaction was observed in between its polyaromatic fragments whose distance is approximately 3.44 Å. The polycyclic subunits overlap each other, being line-symmetrically aligned (Figure 5a). Meanwhile, the isomer **4c** has two independent molecules in the unit cell, and the torsion angles are 67.1° and 106.8°, respectively (Figure 4d and 4f). As displayed in Figure 4b, the aromatic fragments are point-symmetrically overlapped with the π–π stacking distance of around 3.45 Å. It is noteworthy that both **4b** and **4c** pile up while minimizing the steric repulsion between the *tert*-butyl groups which occupy “staggered” orientations in their crystal structures (Figure 5c and 5d). Unfortunately, the crystal structure of **4a** was not determined after numerous attempts for obtaining crystals suitable for the X-ray analysis. Based on these observations, it is reasonable to conclude that the *tert*-butyl substituents effectively restricted the stacking structure to the specific conformations, thereby facilitating the assembly of well-ordered aggregates in the solid state [50–54].

## Conclusion

In summary, we have achieved the synthesis of a series of CPL-active polyheteroaromatic compounds from readily available chiral BINOLs via the *O*-arylation and subsequent palladium-catalyzed C–H/C–H coupling reaction. The substitution pattern on the BBZFPy skeleton had much effect on the solid-state optical properties. Particularly, the compounds **4b** and **4c** bearing terminal *tert*-butyl groups exhibited solid-state fluorescence with the enhanced CPL characteristics (*g*_lum_ = 6.68 × 10^−3^ and 6.06 × 10^−3^), as compared to those in solution. Their solid-state structures were investigated by X-ray diffraction analysis to find well-ordered intermolecular stacking structures within the crystals.

## Experimental

### General

All manipulations were performed under N_2_ using standard Schlenk techniques unless otherwise noted. DMF was dried and deoxygenated by a Glass Counter Solvent Dispending System (Nikko Hansen & Co., Ltd.). DMSO was distilled from CaH_2_ and stored over molecular sieves 4 Å. Silica gel column chromatography was performed using Wakosil^®^ C-200 (64–210 μm). Nuclear magnetic resonance spectra were measured at 400 MHz (^1^H NMR) and at 100 MHz (^13^C NMR) in 5 mm NMR tubes. ^1^H NMR chemical shifts were reported in ppm relative to the resonance of TMS (δ 0.00) or the residual solvent signals at δ 7.26 for CDCl_3_. ^13^C NMR chemical shifts were reported in ppm relative to the residual solvent signals at δ 77.2 for CDCl_3_. Melting points were measured using a Mettler Toledo MP90. High-resolution mass spectra (HRMS) were recorded by APCI-TOF or EI. Preparative gel permeation chromatography (GPC) was conducted with a YMC GPC-T2000 column eluting with CHCl_3_. Absorption spectra were recorded with JASCO V-750 spectrometer. Photoluminescence spectra were recorded with JASCO FP-8500 spectrometer. Quantum yield was determined using an integration sphere system. CD and CPL spectra were recorded with JASCO J-820AC and JASCO CPL-300 spectrometers. HPLC analysis was carried out with JASCO EXTREMA (PU4180/MD4015/CO4065) equipped with YMC CHIRAL ART Amylose-SA and YMC CHIRAL ART Cellulose-SB columns.

### Preparation of 6,6'-di-*tert*-butyl-BINOL (**1**)

Compound **1** was prepared according to a literature procedure [55]. ^1^H NMR (400 MHz, CDCl_3_) δ 1.38 (s, 18H), 4.96 (s, 2H), 7.13 (d, *J* = 8.9 Hz, 2H), 7.36 (d, *J* = 8.9 Hz, 2H), 7.41 (dd, *J* = 2.0, 8.9 Hz, 2H), 7.82 (d, *J* = 2.0 Hz, 2H), 7.94 (d, *J* = 8.9 Hz, 2H); ^13^C NMR (100 MHz, CDCl_3_) δ 31.25, 34.61, 110.62, 117.54, 123.49, 124.01, 126.36, 129.35, 131.34, 131.42, 146.69, 152.29; HRMS–APCI (*m/z*): [M + H]^+^ calcd for C_28_H_31_O_2_, 399.2330; found, 399.2319. The enantiomeric purity was confirmed by HPLC analysis: CHIRAL ART Amylose-SA column, *n*-hexane/2-propanol 90:10, 1.0 mL/min, 40 °C; (*S*)-**1**: *t*_R_ = 17.9 min, (*R*)-**1**: *t*_R_ = 6.83 min, UV detection at 250.0 nm.

### Preparation of **2**

To a 20 mL two-necked round-bottomed flask were added **1** (796 mg, 2.0 mmol) and Cs_2_CO_3_ (978 mg, 6.0 mmol). 2,6-Difluoropyridine (0.55 mL, 6.0 mmol) and DMF (10 mL) were added via syringe. The mixture was stirred at 40 °C for 48 h under N_2_. The resulting mixture was extracted with EtOAc. The organic layer was washed with water, dried over Na_2_SO_4_, and evaporated in vacuo. The residue was purified by silica gel column chromatography (eluent: hexane/EtOAc 2:1) and GPC to give the title compound as white solid; (*S*)-**2** (1.01 g, 86% yield), (*R*)-**2** (1.06 g, 90% yield). Mp 212–214 °C; ^1^H NMR (400 MHz, CDCl_3_) δ 1.37 (s, 18H), 6.28 (dd, *J =* 2.6, 7.8 Hz, 2H), 6.37 (dd, *J* = 1.2, 8.0 Hz, 2H), 7.20 (d, *J =* 0.8 Hz, 2H), 7.33 (d, *J =* 8.8 Hz, 2H), 7.34 (d, *J =* 8.8 Hz, 2H), 7.39 (dd, *J* = 2.0, 8.4 Hz, 2H), 7.77 (d, *J =* 2.0 Hz, 2H), 7.89 (d, *J =* 8.8 Hz, 2H); ^13^C NMR (100 MHz, CDCl_3_) δ 31.18, 34.68, 101.52, 101.87, 121.38, 122.81, 123.20, 125.29, 126.12, 129.51, 131.09, 131.96, 142.60 (d, *J*_C–F_* =* 7.8 Hz), 147.89, 148.95, 161.61 (d, *J*_C–F_* =* 14.0 Hz), 162.28 (d, *J*_C–F_* =* 240 Hz), ^19^F NMR (376 MHz, CDCl_3_) δ 69.06; HRMS–APCI (*m/z*): [M + H]^+^ calcd for C_38_H_35_F_2_N_2_O_2_, 589.2644; found, 589.2661. The enantiomeric purity was confirmed by HPLC analysis: CHIRAL ART Cellulose-SB column, *n*-hexane/chloroform 95:5, 1.0 mL/min, 40 °C; (*S*)-**2**: *t*_R_ = 9.86 min, (*R*)-**2**: *t*_R_ = 21.29 min, UV detection at 250.0 nm.

### Preparation of **3a–c**

**Compound 3a:** To a 10 mL Schlenk flask were added **2** (294 mg, 0.5 mmol), phenol (103 mg, 1.1 mmol), and Cs_2_CO_3_ (358 mg, 1.1 mmol). DMSO (3.5 mL) was added via the syringe. The mixture was stirred at 100 °C for 18 h under N_2_. The resulting mixture was extracted with EtOAc. The organic layer was washed with water, dried over Na_2_SO_4_, and evaporated in vacuo. The residue was purified by silica gel column chromatography (eluent: hexane/EtOAc 6:1) and GPC to give the title compound as white solid; (*S*)-**3a** (309 mg, 84% yield), (*R*)-**3a** (332 mg, 90%). Mp 110–112 °C; ^1^H NMR (400 MHz, CDCl_3_) δ 1.29 (s, 18H), 6.10 (d, *J* = 7.9 Hz, 2H), 6.16 (d, *J* = 7.9 Hz, 2H), 6.91 (dd, *J* = 0.92, 8.5 Hz, 4H), 6.99–7.03 (m, 4H), 7.14–7.17 (m, 8H), 7.22 (d, *J* = 8.8 Hz, 2H), 7.68 (d, *J* = 2.0 Hz, 2H), 7.73 (d, *J* = 8.8 Hz, 2H); ^13^C NMR (100 MHz, CDCl_3_) δ 31.23, 34.63, 103.72, 104.83, 121.03, 121.55, 122.67, 123.15, 124.27, 125.03, 126.13, 129.10, 129.30, 130.90, 132.04, 141.35, 147.49, 149.54, 154.00, 161.86, 162.48; HRMS–APCI (*m/z*): [M + H]^+^ calcd for C_50_H_45_N_2_O_4_, 737.3367; found, 737.3374. The enantiomeric purity was confirmed by HPLC analysis: CHIRAL ART Amylose-SA column, *n*-hexane/chloroform 95:5, 1.0 mL/min, 40 °C; (*S*)-**3a**: *t*_R_ = 7.25 min, (*R*)-**3a**: *t*_R_ = 14.12 min, UV detection at 250.0 nm.

**Compound 3b:** Synthesized similarly to **3a** using 4-*tert*-butylphenol. Purified by silica gel column chromatography (eluent: hexane/EtOAc 4:1) and GPC to give the title compound as white solid; (*S*)-**3b** (399 mg, 94% yield), (*R*)-**3b** (386 mg, 91% yield). Mp 113–115 °C; ^1^H NMR (400 MHz, CDCl_3_) δ 1.16 (s, 18H), 1.35 (s, 18H), 6.20 (d, *J* = 8.0 Hz, 2H), 6.28 (d, *J* = 7.6 Hz, 2H), 6.77 (d, *J* = 8.9 Hz, 2H), 6.88 (dd, *J* = 2.1, 6.7 Hz, 4H), 7.09 (dd, *J* = 2.0, 8.0 Hz, 2H), 7.12 (dd, *J* = 2.1, 7.0 Hz, 4H), 7.23 (d, *J* = 8.8 Hz, 2H), 7.29 (t, *J* = 8.0 Hz, 2H), 7.72 (d, *J* = 2.0 Hz, 2H), 7.78 (d, *J* = 8.8 Hz, 2H); ^13^C NMR (100 MHz, CDCl_3_) δ 31.23, 31.95, 34.22, 34.58, 103.43, 104.29, 120.83, 121.94, 122.40, 123.40, 125.01, 125.73, 126.23, 128.83, 130.79, 132.05, 141.29, 146.89, 147.26, 149.60, 151.15, 161.82, 162.41; HRMS–APCI (*m/z*): [M + H]^+^ calcd for C_58_H_61_N_2_O_4_, 849.4603; found, 849.4626. The enantiomeric purity was confirmed by HPLC analysis: CHIRAL ART Amylose-SA column, *n*-hexane/chloroform 95:5, 1.0 mL/min, 40 °C; (*S*)-**3b**: *t*_R_ = 8.36 min, (*R*)-**3b**: *t*_R_ = 10.05 min, UV detection at 250.0 nm.

**Compound 3c:** Synthesized similarly to **3a** using 3-*tert*-butylphenol. Purified by silica gel column chromatography (eluent: hexane/EtOAc 6:1) and GPC to give the title compound as white solid; (*S*)**-3c** (377 mg, 89% yield), (*R*)**-3c** (403 mg, 95% yield). Mp 88–90 °C; ^1^H NMR (400 MHz, CDCl_3_) δ 1.17 (s, 18H), 1.36 (s, 18H), 6.14 (d, *J* = 8.0 Hz, 2H), 6.23 (d, *J* = 7.6 Hz, 2H), 6.79 (ddd, *J* = 1.0, 2.2, 7.3 Hz, 2H), 7.02 (t, *J* = 2.0 Hz, 2H), 7.09–7.13 (m, 4H), 7.16–7.28 (m, 8H), 7.73 (d, *J* = 1.6 Hz, 2H), 7.76 (d, *J* = 8.8 Hz, 2H); ^13^C NMR (100 MHz, CDCl_3_) δ 31.16, 31.22, 34.61, 34.63, 103.53, 104.62, 117.99, 118.29, 121.39, 121.53, 122.67, 123.11, 124.94, 126.13, 128.82, 129.06, 130.86, 132.03, 141.26, 147.42, 149.48, 153.01, 153.87, 162.23, 162.50; HRMS–APCI (*m/z*): [M + H]^+^ calcd for C_58_H_61_N_2_O_4_, 849.4599; found, 849.4626. The enantiomeric purity was confirmed by HPLC analysis: CHIRAL ART Amylose-SA column, *n*-hexane/chloroform 95:5, 1.0 mL/min, 40 °C; (*S*)-**3c**: *t*_R_ = 9.00 min, (*R*)-**3c**: *t*_R_ = 8.61 min, UV detection at 250.0 nm.

### Preparation of **4a–c**

**Compound 4a:** To a 10 mL Schlenk flask were added **3a** (184 mg, 0.25 mmol), Pd(TFA)_2_ (24.9 mg, 0.075 mmol), AgOAc (167 mg, 1.0 mmol), and PivOH (2.0 mL). The mixture was heated at 150 °C for 24 h under air. After cooling to room temperature, the resulting mixture was diluted with water and filtered through a pad of Celite eluting with dichloromethane. The filtrate was washed with water, dried over Na_2_SO_4_, and concentrated in vacuo. The obtained crude material was again subjected to the catalytic conditions described above. The residue was purified by silica gel column chromatography (eluent: hexane/dichloromethane 4:1) and GPC to give the title compound as pale yellow solid; (*S*)-**4a** (28.2 mg, 16% yield), (*R*)-**4a** (18.1 mg, 10% yield). Mp >300 °C; ^1^H NMR (400 MHz, CDCl_3_) δ 1.46 (s, 18H), 7.44–7.52 (m, 8H), 7.62 (d, *J* = 8.0 Hz, 2H), 8.07 (dd, *J* = 0.8, 7.6 Hz, 2H), 8.12 (d, *J* = 2.0 Hz, 2H), 8.67 (s, 2H), 8.96 (s, 2H); ^13^C NMR (100 MHz, CDCl_3_) δ 31.25, 34.82, 112.20, 112.62, 113.07, 113.65, 120.13, 120.59, 122.80, 122.93, 123.21, 123.61, 123.62, 125.68, 125.81, 127.39, 130.67, 130.90, 147.553, 151.62, 154.63, 161.90, 163.10; HRMS–APCI (*m/z*): [M + H]^+^ calcd for C_50_H_37_N_2_O_4_, 729.2723; found, 729.2748. The enantiomeric purity was determined by HPLC analysis: CHIRAL ART Amylose-SA column, *n*-hexane/chloroform 60:40, 1.0 mL/min, 40 °C; (*S*)-**4a**: *t*_R_ = 4.39 min, (*R*)-**4a**: *t*_R_ = 6.73 min, UV detection at 250.0 nm.

**Compound 4b:** Synthesized similarly to **4b** from **3b** (254 mg, 0.30 mmol). Purified by silica gel column chromatography (eluent: hexane/EtOAc 4:1) and GPC to give the title compound as pale yellow solid; (*S*)-**4b** (45.4 mg, 18% yield), (*R*)-**4b** (30.2 mg, 12% yield). Single crystals suitable for the X-ray analysis were obtained by slow evaporation from EtOAc solution. Mp >300 °C; ^1^H NMR (400 MHz, CDCl_3_) δ 1.46 (s, 18H), 1.48 (s, 18H), 7.47–7.71 (m, 8H), 8.07 (d, *J* = 0.8 Hz, 2H), 8.11 (d, *J* = 1.6 Hz, 2H), 8.65 (s, 2H), 8.98 (s, 2H); ^13^C NMR (100 MHz, CDCl_3_) δ 31.26, 31.87, 34.82, 34.98, 111.49, 112.62, 113.40, 113.47, 116.98, 119.97, 122.31, 122.75, 122.35, 123.57, 125.13, 125.69, 125.73, 130.64, 130.87, 146.82, 147.48, 151.62, 152.78, 162.25, 162.94; HRMS–EI (*m/z*): [M]^+^ calcd for C_58_H_52_N_2_O_4_, 840.3927; found, 840.3932; 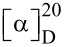 = +31.8 (*S*-isomer), −32.4 (*R*-isomer) as CHCl_3_ solution. The enantiomeric purity was determined by HPLC analysis: CHIRAL ART Amylose-SA column, *n*-hexane/2-propanol 90:10, 1.0 mL/min, 40 °C; (*S*)-**4b**: *t*_R_ = 15.95 min, (*R*)-**4b**: *t*_R_ = 24.38 min, UV detection at 250.0 nm.

**Compound 4c:** Synthesized similarly to **4c** from **3c** (254 mg, 0.3 mmol). Purified by silica gel column chromatography (eluent: hexane/EtOAc 4:1) and GPC to give the title compound as pale yellow solid; (*S*)-**4c** (74.1 mg, 29% yield), (*R*)-**4c** (90.0 mg, 36% yield). Single crystals suitable for the X-ray analysis were obtained by hexane vapor diffusion into CHCl_3_ solution. Mp >300 °C; ^1^H NMR (400 MHz, CDCl_3_) δ 1.41 (s, 18H), 1.45 (s, 18H), 7.47–7.64 (m, 6H), 7.637 (d, *J* = 0.8 Hz, 2H), 7.97 (d, *J* = 8.4 Hz, 2H), 8.10 (d, *J* = 0.8 Hz ,2H), 8.65 (s, 2H), 8.91 (s, 2H); ^13^C NMR (100 MHz, CDCl_3_) δ 31.26, 31.57, 34.81, 3.38, 109.04, 112.62, 113.23, 113.39, 119.98, 119.99, 120.05, 121.20, 122.53, 123.35, 123.58, 125.69, 125.70, 130.63, 130.88, 147.46, 151.62, 151.76, 155.05, 162.12, 162.77; HRMS–APCI (*m/z*): [M + H]^+^ calcd for C_58_H_53_N_2_O_4_, 841.3970; found, 841.4000. The enantiomeric purity was determined by HPLC analysis: CHIRAL ART Amylose-SA column, *n*-hexane/chloroform 70:30, 1.0 mL/min, 40 °C; (*S*)-**4c**: *t*_R_ = 4.48 min, (*R*)-**4c**: *t*_R_ = 6.66 min, UV detection at 250.0 nm.

### Preparation of **5**

In a 200 mL three-necked round-bottomed flask, **1** (1.99 g, 5.0 mmol) was added to a suspension of *t-*BuOK (1.40 g, 12.5 mmol) in THF (80 mL) at 0 °C. After stirring for 2 h, Ph_2_IOTf (5.38 g, 12.5 mmol) was added in one portion. The mixture was allowed to warm to 40 °C, and stirred at this temperature until the complete consumption **1** was confirmed by TLC. The resulting suspension was poured into ice water and extracted with Et_2_O. The combined organic layer was dried over Na_2_SO_4_ and concentrated in vacuo. The residue was purified by silica gel column chromatography (eluent: hexane/EtOAc 20:1) and GPC (CHCl_3_) to give the title compound as white solid; (*S*)-**5** (1.59 g, 58% yield), (*R*)-**5** (1.71 g, 62% yield). Mp 167.0–169.0 °C; ^1^H NMR (400 MHz, CDCl_3_) δ 1.39 (s, 18H), 6.79 (d, *J* = 7.6, Hz, 4H), 6.92 (t, *J* = 7.6 Hz, 2H), 7.10 (t, *J* = 7.6 Hz, 4H), 7.17 (d, *J* = 8.9 Hz, 2H), 7.27 (d, *J* = 9.2 Hz, 2H), 7.39 (dd, *J* = 2.0, 8.9 Hz, 2H), 7.79 (d, *J* = 1.7 Hz, 2H), 7.83 (d, *J* = 8.9 Hz, 2H); ^13^C NMR (100 MHz, CDCl_3_) δ 31.25, 34.65, 118.84, 119.29, 122.06, 122.43, 123.04, 125.46, 125.63, 129.19 129.46, 130.30, 132.44, 147.18, 152.08, 157.74; HRMS–APCI [*m/z*]: [M + H]^+^ calcd for C_40_H_39_O_2_, 551.2959; found, 551.2945. The enantiomeric purity was confirmed by HPLC analysis: CHIRAL ART Amylose-SA column, *n*-hexane/chloroform 98:2, 1.0 mL/min, 40 °C; (*S*)-**5**: *t*_R_ = 4.91 min, (*R*)-**5**: *t*_R_ = 5.32 min, UV detection at 250.0 nm.

### Preparation of **6**

To a 10 mL Schlenk flask were added **5** (165 mg, 0.3 mmol), Pd(TFA)_2_ (29.9 mg, 0.09 mmol), AgOAc (198 mg, 1.2 mmol), and PivOH (3.0 mL). The mixture was heated at 150 °C for 24 h under air. After cooling to room temperature, the resulting mixture was diluted with water and filtered through a pad of Celite eluting with dichloromethane. The filtrate was washed with water, dried over Na_2_SO_4_, and concentrated in vacuo. The residue was purified by silica gel column chromatography (eluent: hexane/EtOAc 2:1) and GPC to give the title compound as pale yellow solid; (*S*)-**6** (30.1 mg, 18% yield), (*R*)-**6** (30.3 mg, 18% yield). Mp 212–214 °C; ^1^H NMR (400 MHz, CDCl_3_) δ 1.41 (s, 18H), 7.31 (dd, *J* = 0.8, 7.6 Hz, 2H), 7.35 (dd, *J* = 1.2, 7.6 Hz, 2H), 7.38–7.43 (m, 6H), 8.09 (s, 2H), 8.13–8.16 (m, 2H), 8.61 (s, 2H); ^13^C NMR (100 MHz, CDCl_3_) δ 31.26, 34.74, 111.88, 112.25, 119.71, 121.16, 122.62, 123.61, 124.35, 125.08, 125.26, 125.55, 128.08, 130.54, 130.75, 146.80, 153.36, 157.70; HRMS–APCI (*m/z*): [M + H]^+^ calcd for C_40_H_35_O_2_, 547.2622; found, 547.2632. The enantiomeric purity was confirmed by HPLC analysis: CHIRAL ART Amylose-SA column, *n*-hexane/EtOAc 95:5, 1.0 mL/min, 40 °C; (*S*)-**6**: *t*_R_ = 6.47 min, (*R*)-**6**: *t*_R_ = 6.24 min, UV detection at 250.0 nm.

## Supporting Information

File 1 Summary of X-ray crystallography data, copy of NMR spectra, and copy of HPLC charts.
