# Supplementary material for: Synthesis and circularly polarized luminescence properties of BINOL-derived bisbenzofuro[2,3-b:3’,2’-e]pyridines (BBZFPys)
Source: Beilstein J Org Chem. 2020 Mar 6;16:325–36. doi: 10.3762/bjoc.16.32 (PMC7082704; doi:10.3762/bjoc.16.32)
Supplement: File 1 — Summary of X-ray crystallography data, copy of NMR spectra, and copy of HPLC charts. [file Beilstein_J_Org_Chem-16-325-s001.pdf]

BEILSTEIN JOURNAL OF ORGANIC CHEMISTRY

Supporting Information

for

Synthesis and circularly polarized luminescence properties of BINOL-derived bisbenzofuro[2,3-*b*:3’,2’-*e*]pyridines (BBZFPys)

Ryo Takishima, Yuji Nishii, Tomoaki Hinoue, Yoshitane Imai and Masahiro Miura

Beilstein J. Org. Chem. **2020,** *16,* 325–336. doi:10.3762/bjoc.16.32

Summary of X-ray crystallography data, copy of NMR spectra, and copy of HPLC charts

License and Terms: This is a supporting information file under the terms of the Creative Commons Attribution License (https://creativecommons.org/licenses/by/4.0). Please note that the reuse, redistribution and reproduction in particular requires that the authors and source are credited.
The license is subject to the *Beilstein Journal of Organic Chemistry* terms and conditions: (https://www.beilstein-journals.org/bjoc)

Contents

1. Summary of X-ray crystallography data S2

2. Excitation spectra and fluorescence lifetime measurement of **4b** S4

3. Copies of NMR spectra S5

4. Copies of HPLC charts S15

5. References S24

1. Summary of X-ray crystallography data

The structures were refined on F^2 by full-matrix least-squares method, using SHELXL-2016/6.[1] Hydrogen atoms were included in the refinement on calculated positions riding on their carrier atoms. ORTEP-3[2] programs were used to draw the molecules. The CCDC numbers are 1971471 for (*R*)-**4b** and 1971470 for (*R*)-**4c**.

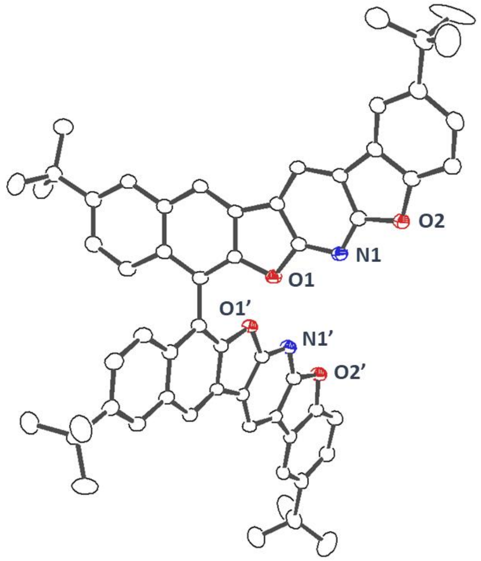

Figure S1. ORTEP drawing for **4b** with 50% thermal ellipsoid. Hydrogen atoms and solvent molecules are omitted for clarity.

Table S1. Crystal data for **4b**

Crystal system	tetragonal
Space group	$P4_322$ (No. 95)
Unit cell parameter [Å, deg]	a = 13.66760(10)
	b = 13.66760(10)
	c = 27.1246(3)
Z	8
R factor ($I > 2.0\sigma(I)$)	$R1 = 0.0746$, $wR2 = 0.2137$
R factor (all data)	$R1 = 0.0777$, $wR2 = 0.2192$
Goodness of fit	1.042
The number of unique reflections	4820 (Rint = 0.0567)
Flack parameter	0.07(9)

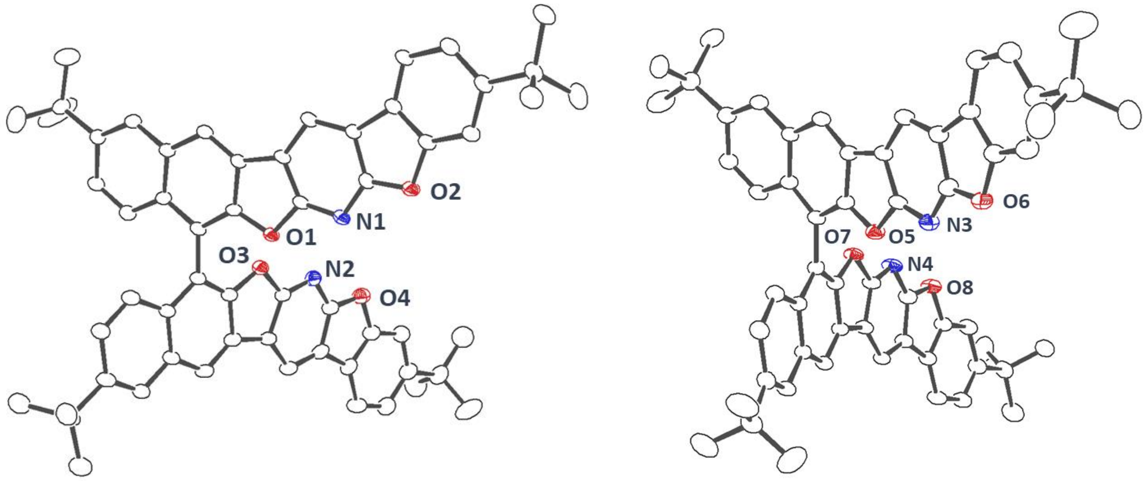

Figure S2. ORTEP drawings for **4c** with 50% thermal ellipsoid. Hydrogen atoms and solvent molecules are omitted for clarity.

Table S2. Crystal data for **4c**

Crystal system	monoclinic
Space group	$P2_1$ (No. 4)
Unit cell parameter [Å, deg]	a = 12.9749(2)
	b = 31.4284(4), β = 99.6540(10)
	c = 14.5186(2)
Z	2
R factor ($I > 2.0\sigma(I)$)	$R1$ = 0.0607, $wR2$ = 0.1774
R factor (all data)	$R1$ = 0.0655, $wR2$ = 0.1845
Goodness of fit	1.080
The number of unique reflections	20761 (Rint = 0.0808)
Flack parameter	0.180(15)

2. Excitation spectra and fluorescence lifetime measurement of 4b

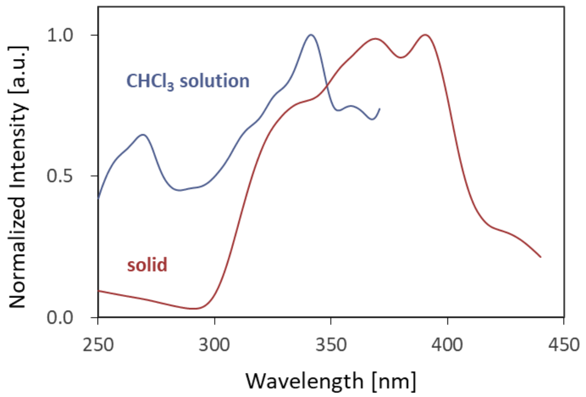

Figure S3. Excitation spectra of **4b** measured as diluted (1.0×10^{-5} M) $CHCl_3$ solution (blue, λ_{flu} 391 nm) and in the solid state (red, λ_{flu} 488 nm).

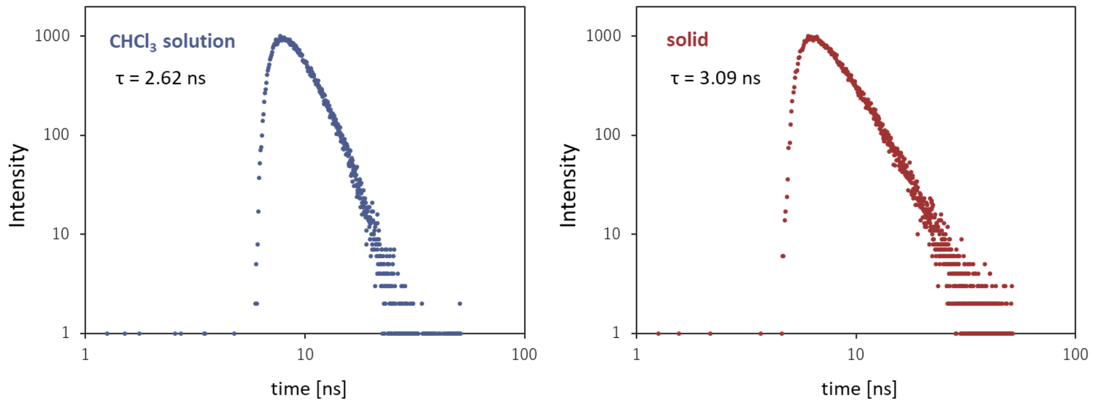

Figure S4. Fluorescence life time measurement of **4b** as diluted (1.0×10^{-5} M) $CHCl_3$ solution (blue, left, λ_{flu} 391 nm) and in the solid state (red, right, λ_{flu} 488 nm).

3. Copies of NMR spectra

[^{1}H, ^{13}C, and ^{19}F NMR Spectra of **2**]

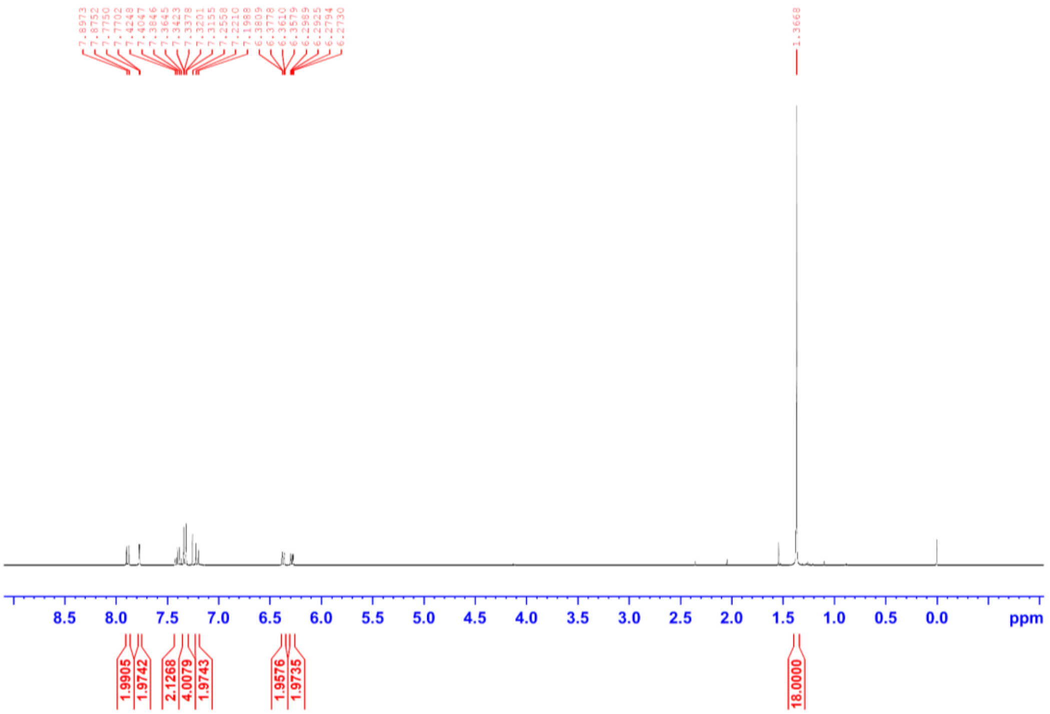

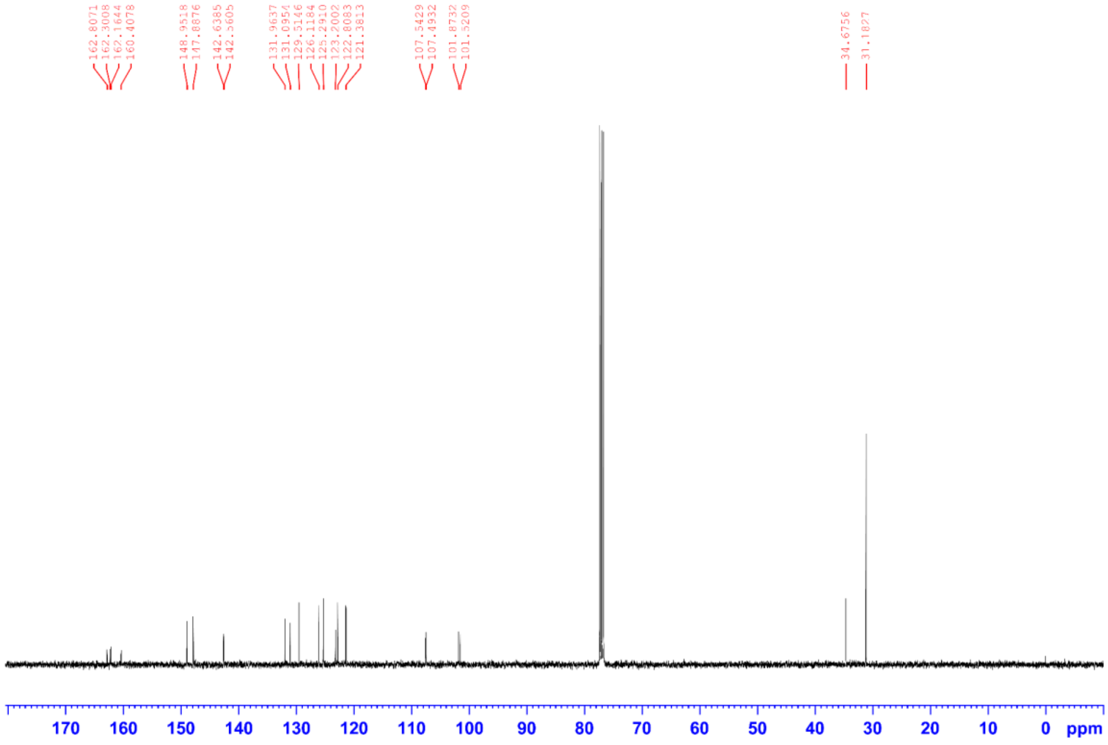

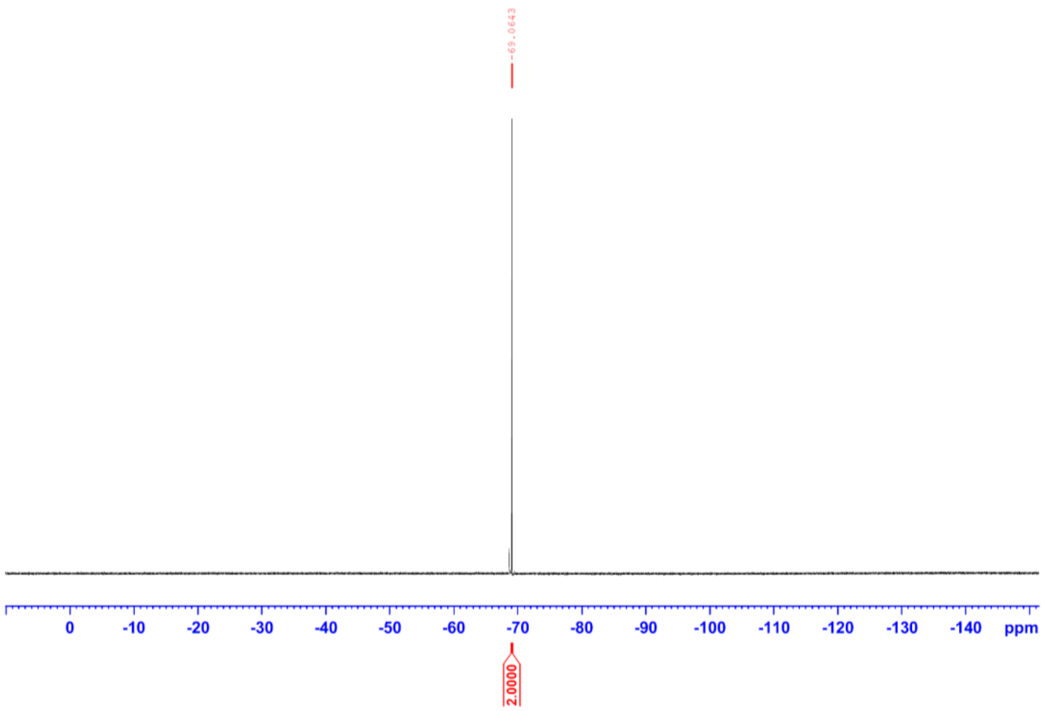
-69.0643
0
-10
-20
-30
-40
-50
-60
-70
-80
-90
-100
-110
-120
-130
-140
ppm
2.0000

[^{1}H and ^{13}C NMR Spectra of **3a**]

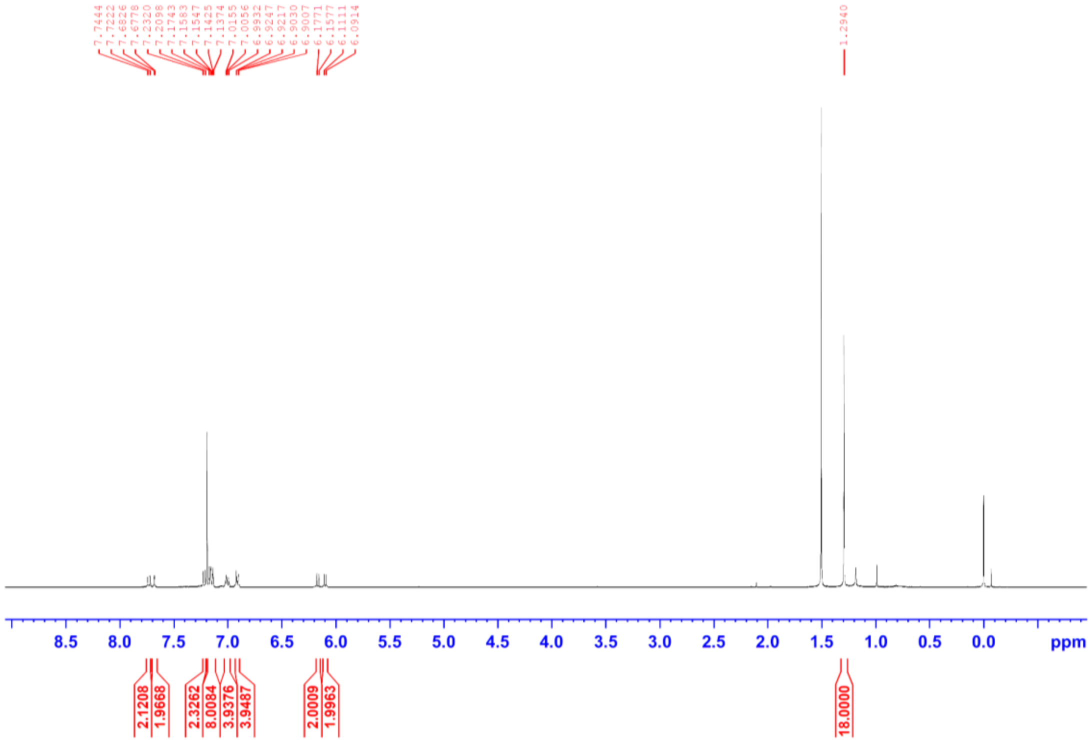

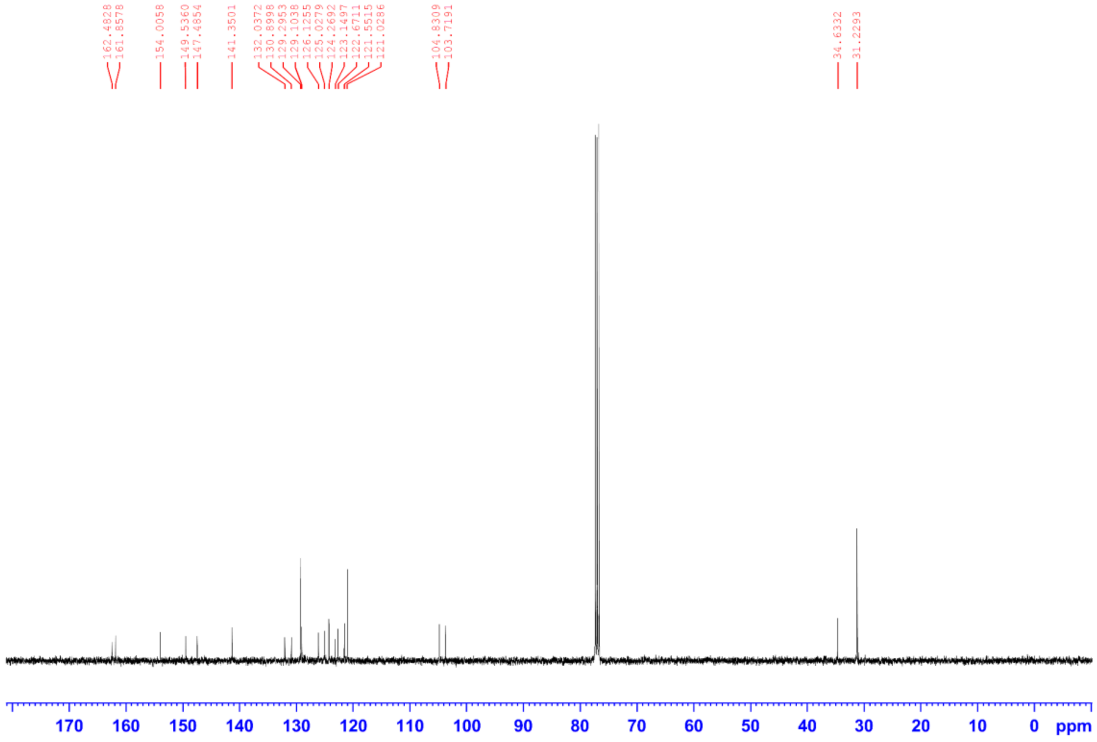

[^{1}H and ^{13}C NMR Spectra of **3b**]

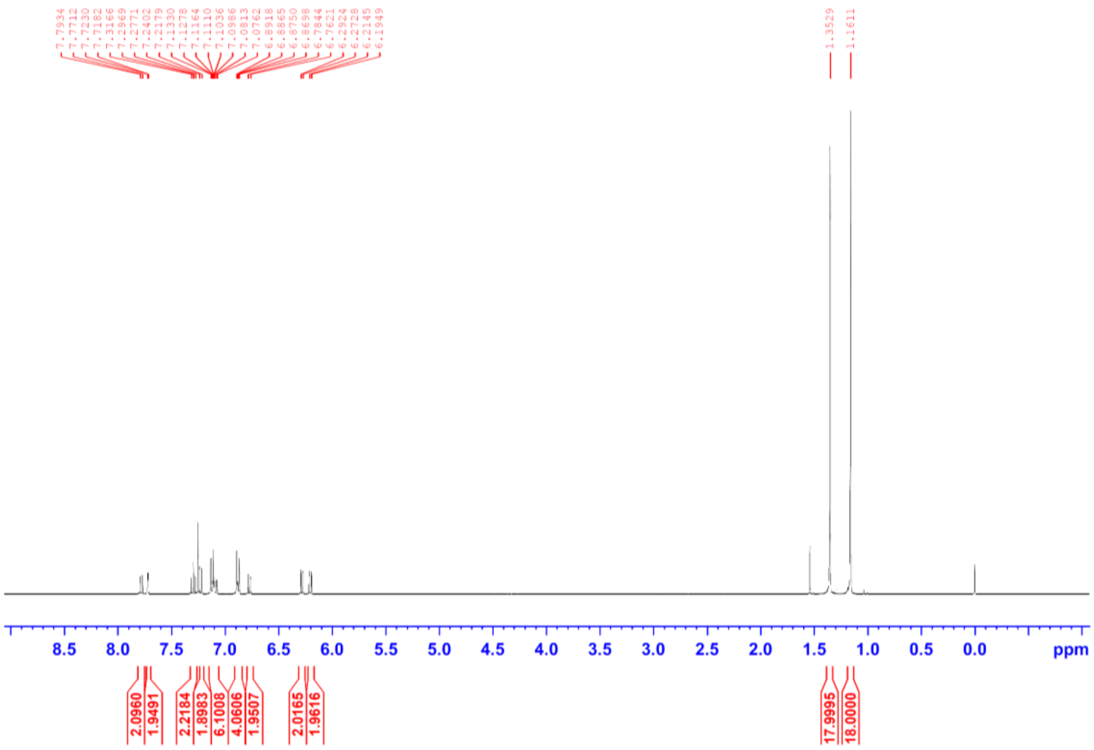

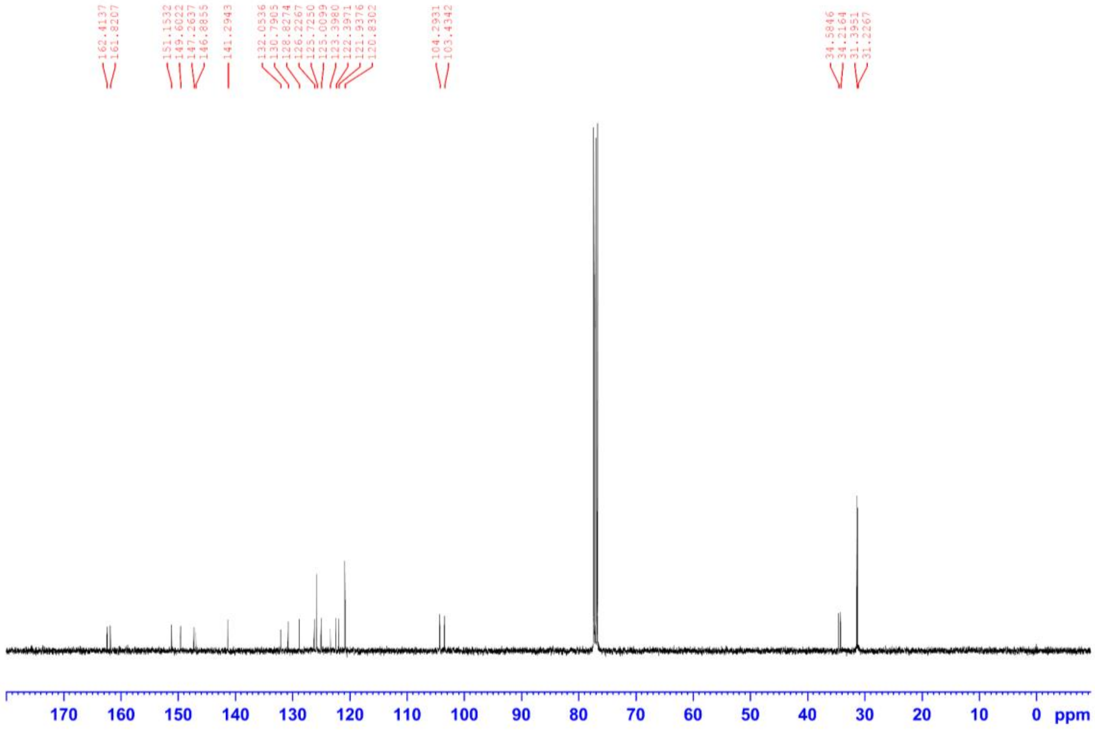

[^{1}H and ^{13}C NMR Spectra of **3c**]

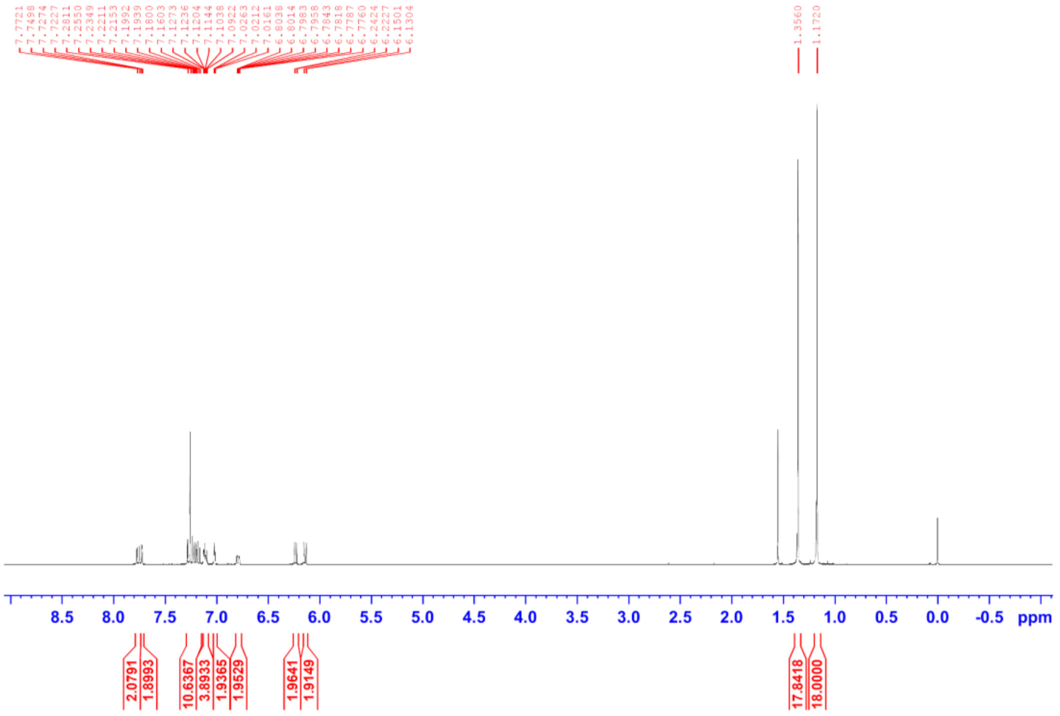

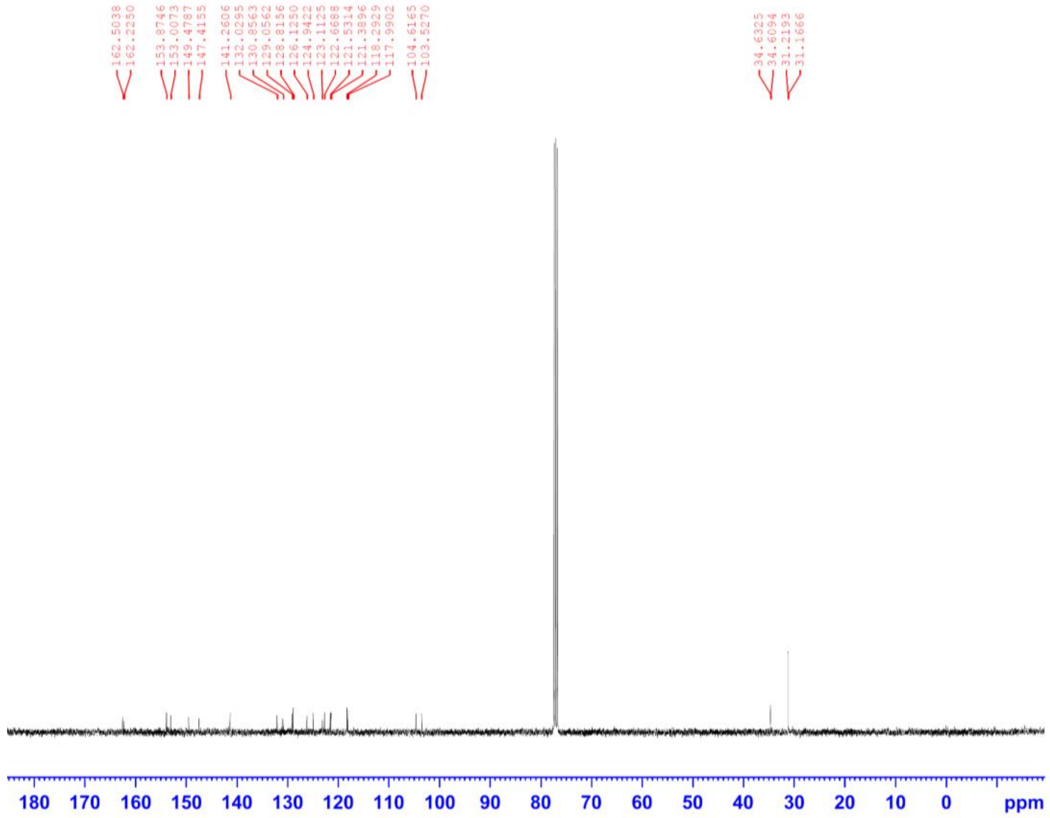

[^{1}H and ^{13}C NMR Spectra of **4a**]

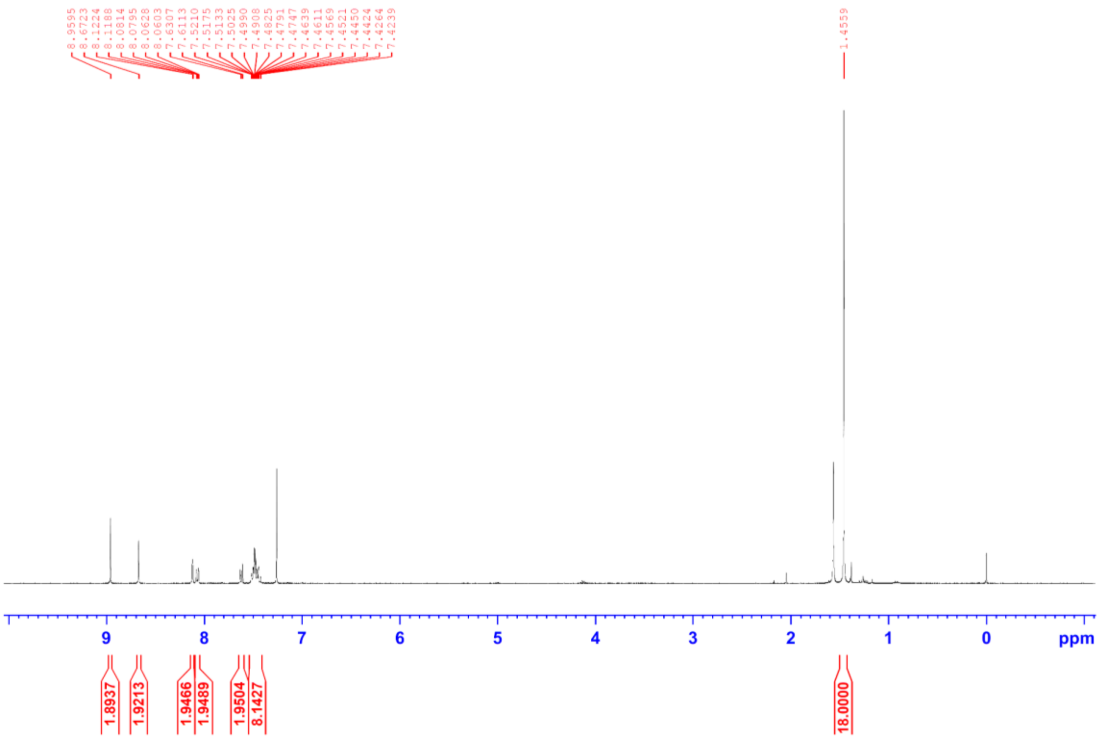

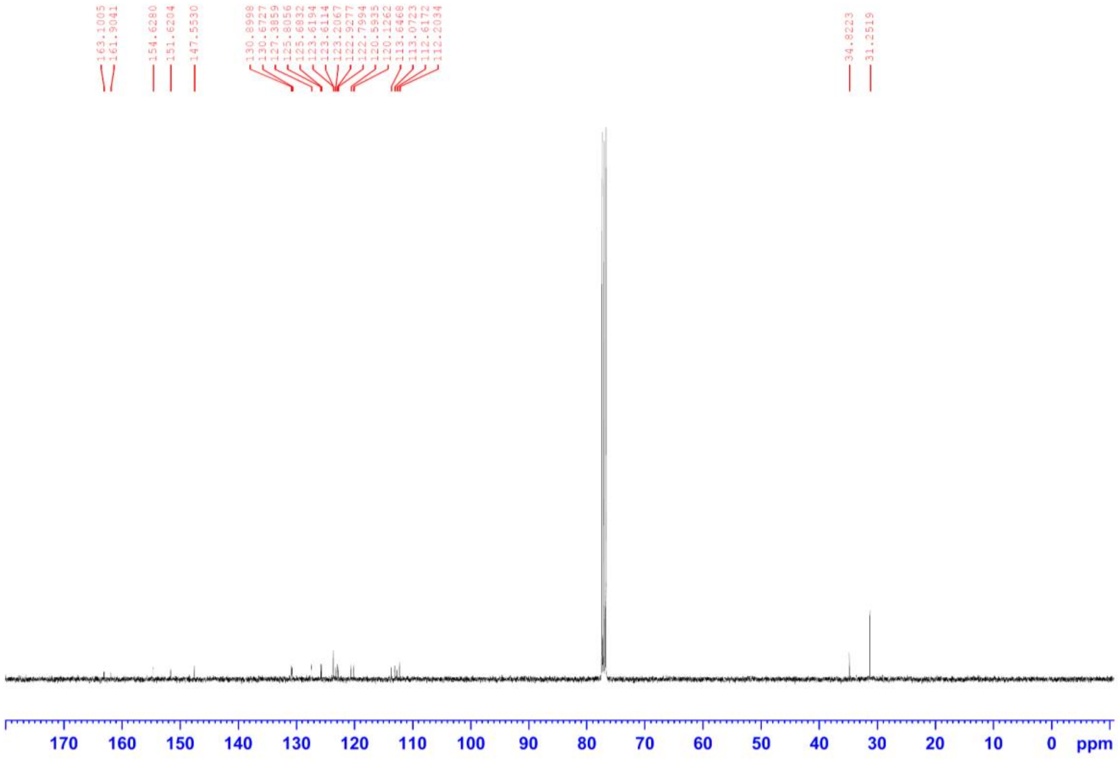

[^{1}H and ^{13}C NMR Spectra of **4b**]

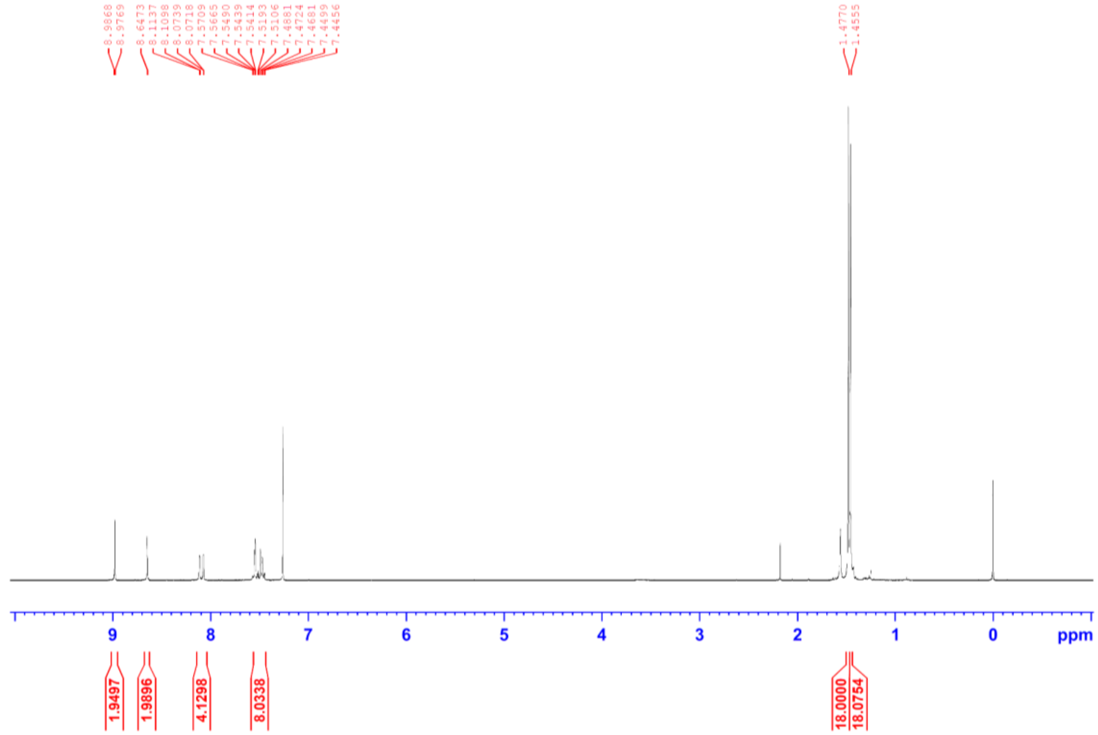

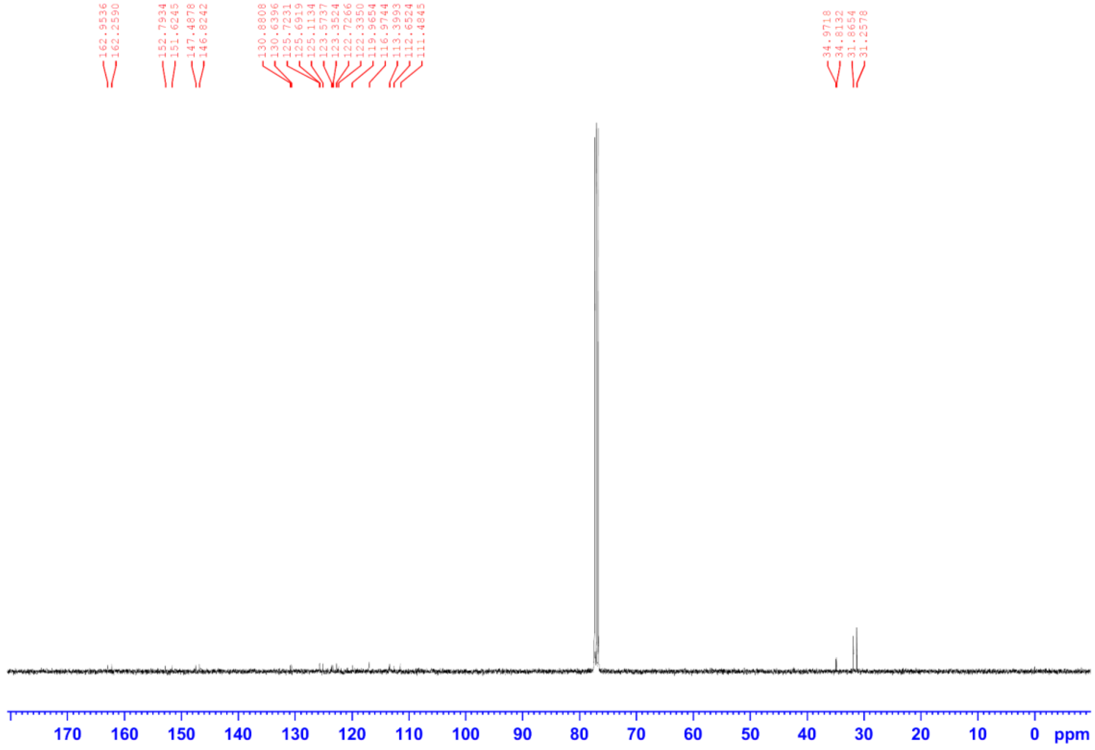

[^{1}H and ^{13}C NMR Spectra of **4c**]

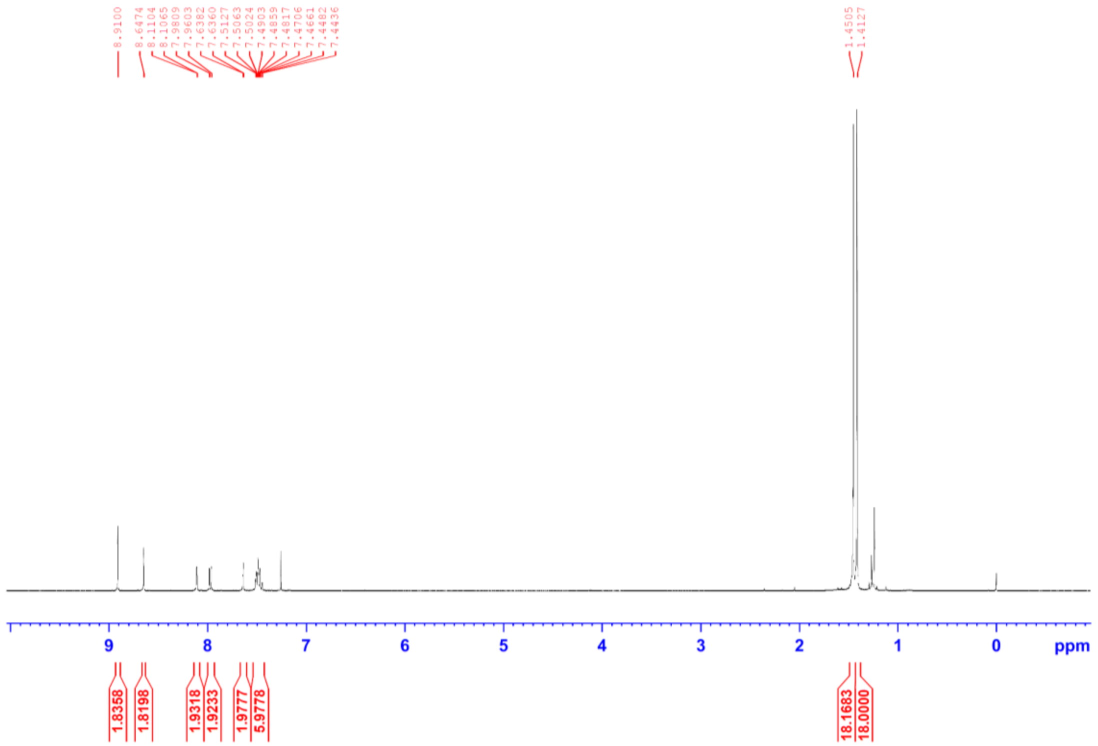

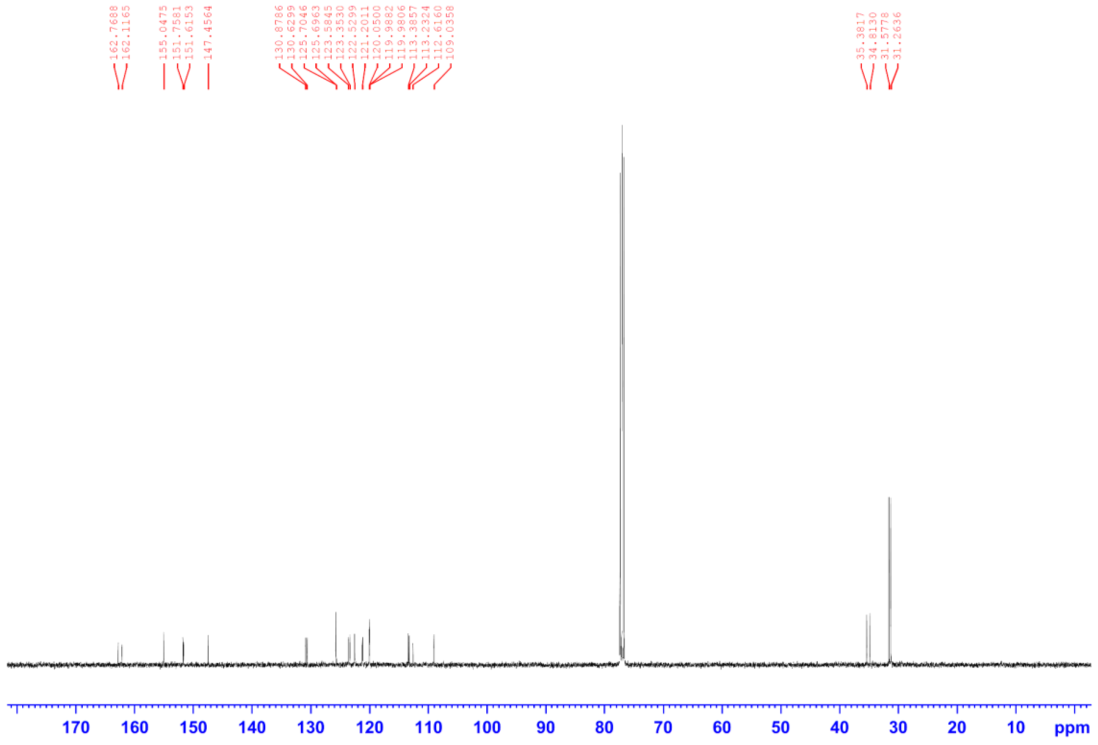

[^{1}H and ^{13}C NMR Spectra of **5**]

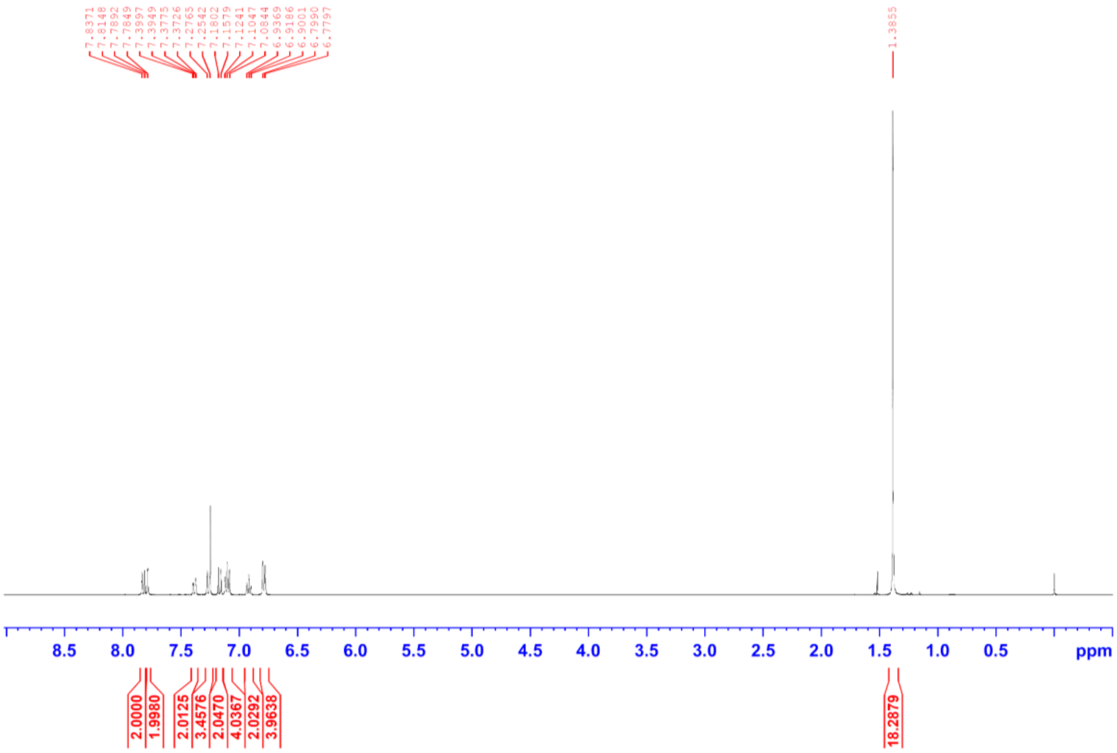

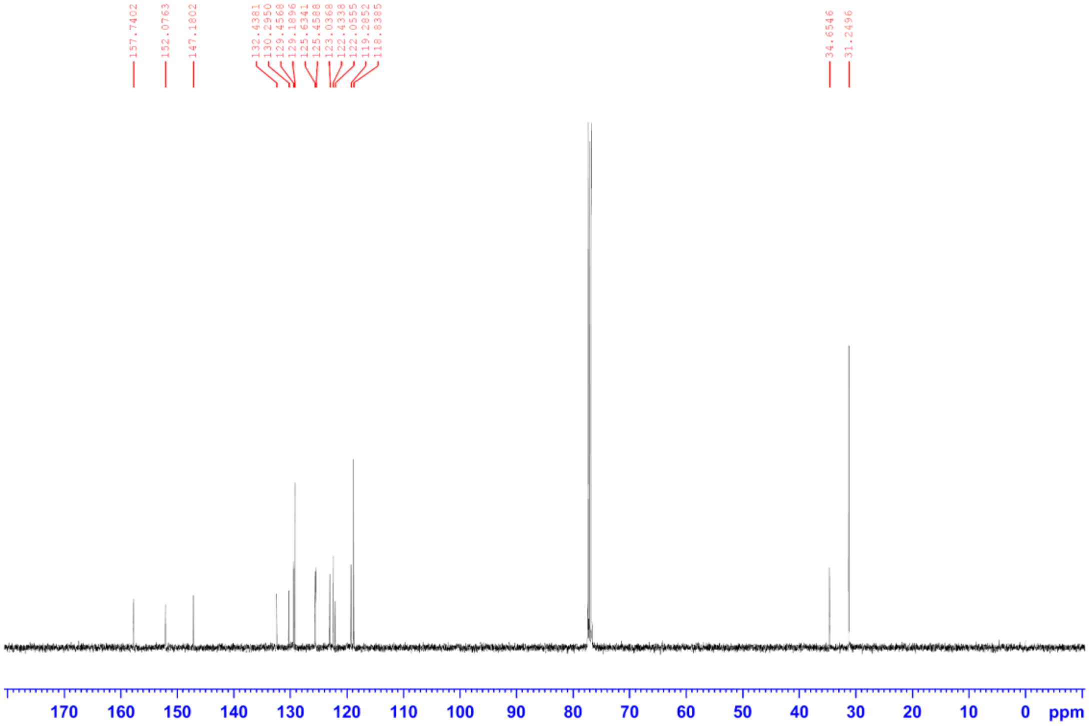

[^{1}H and ^{13}C NMR Spectra of **6**]

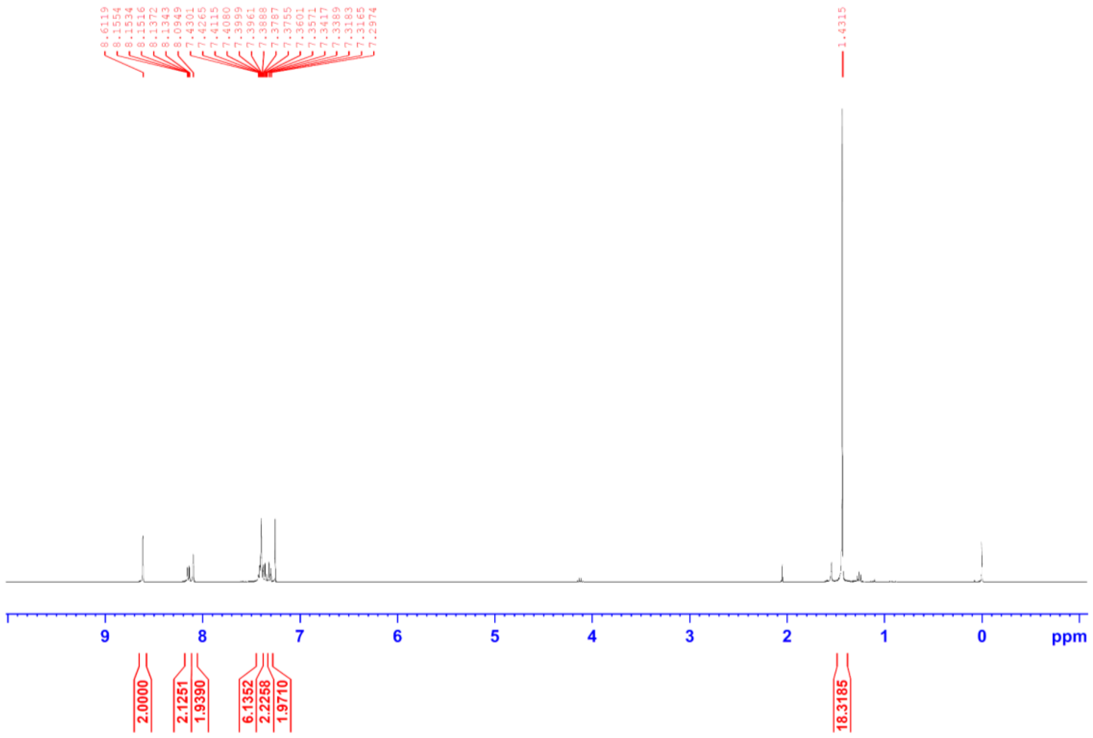

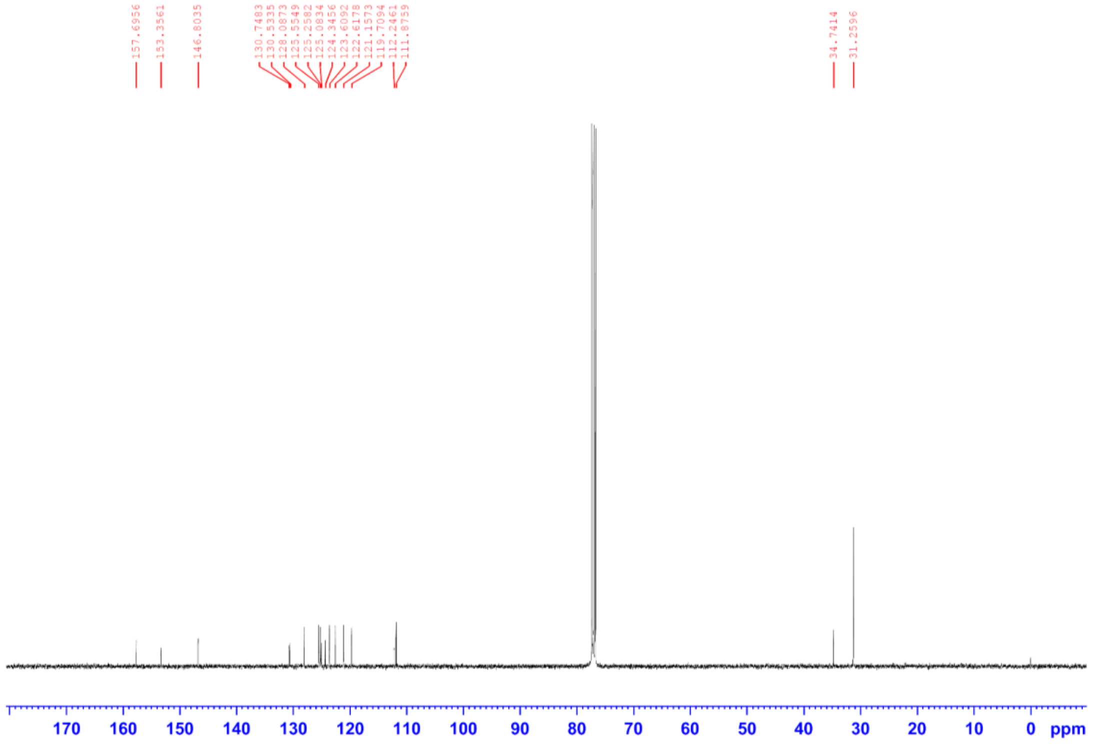

4. Copies of HPLC charts

[(*S*)-**2** and (*R*)-**2**]

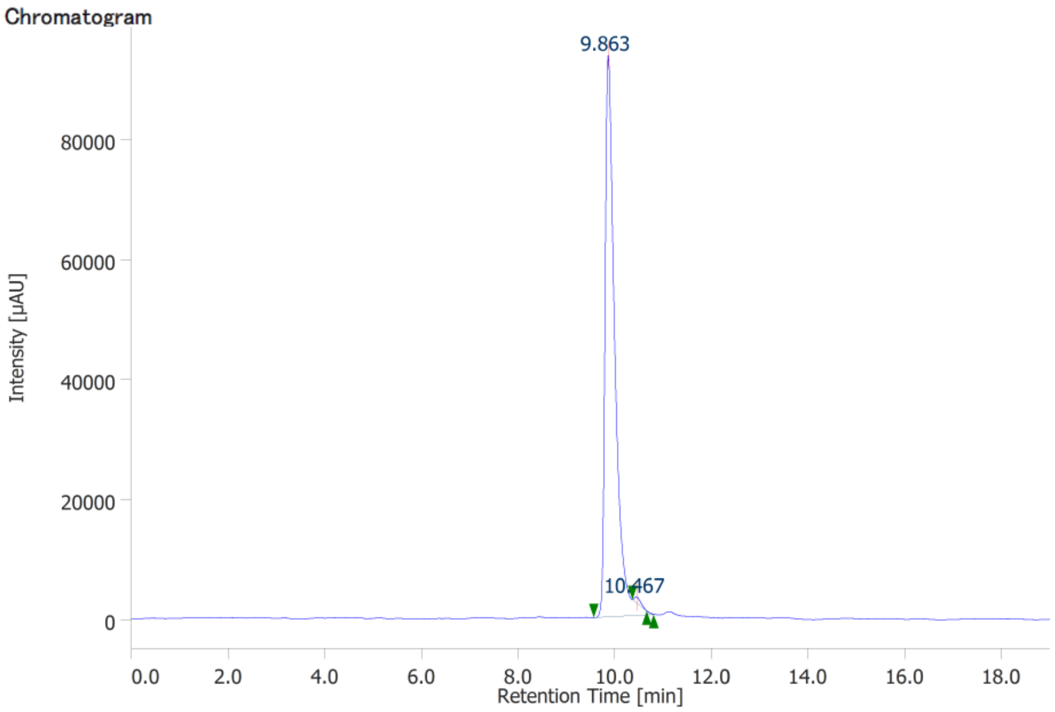

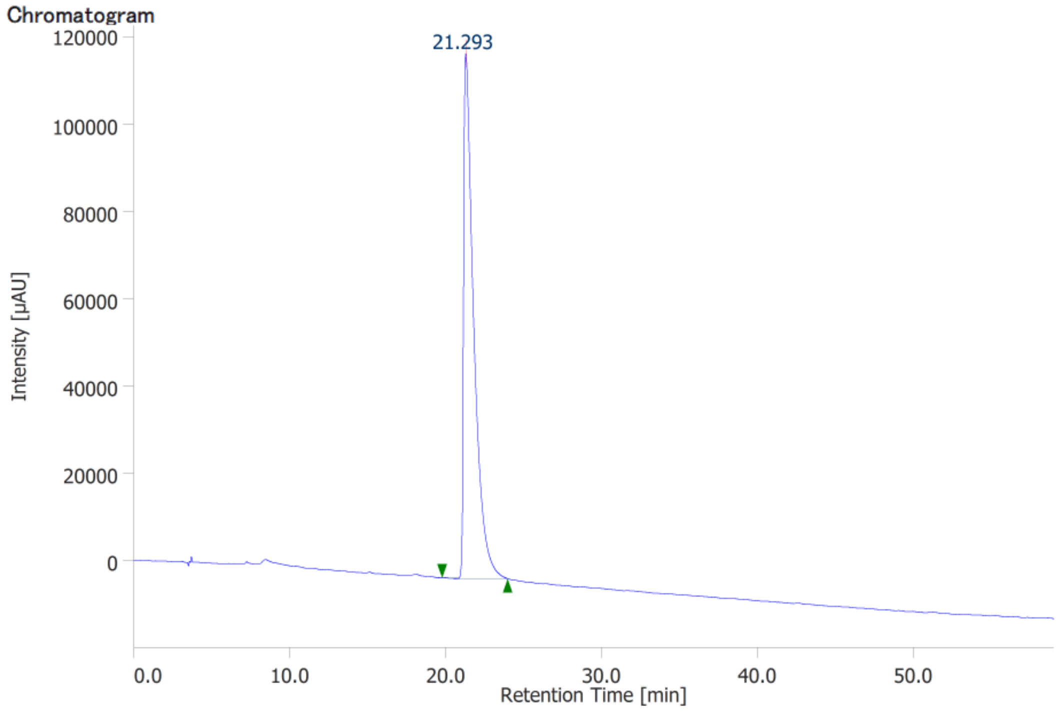

[(*S*)-**3a** and (*R*)-**3a**]

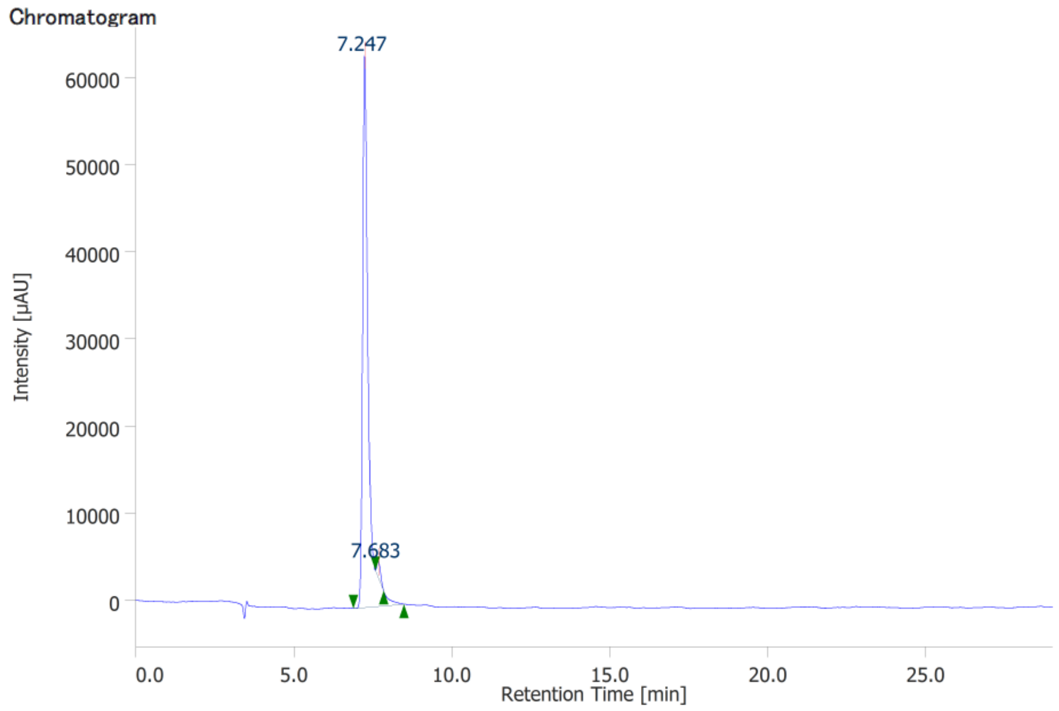
Chromatogram
7.247
7.683
60000
50000
40000
30000
20000
10000
0
Intensity [µAU]
0.0
5.0
10.0
15.0
20.0
25.0
Retention Time [min]

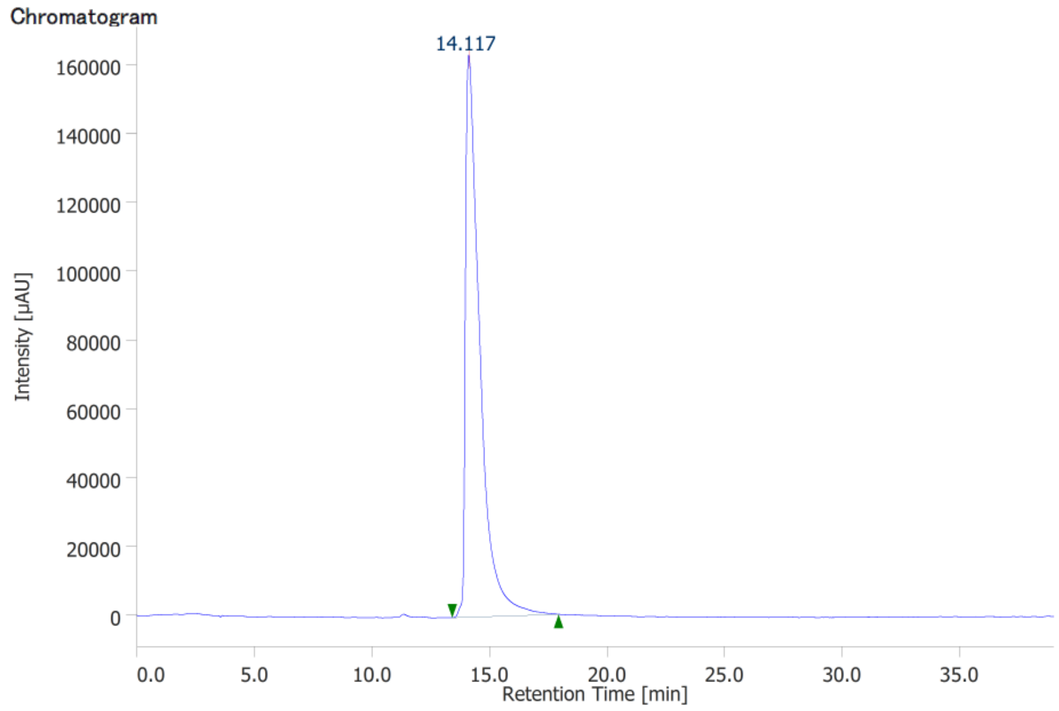
Chromatogram
14.117
160000
140000
120000
100000
80000
60000
40000
20000
0
Intensity [µAU]
0.0
5.0
10.0
15.0
20.0
25.0
30.0
35.0
Retention Time [min]

[(*S*)-**3b** and (*R*)-**3b**]

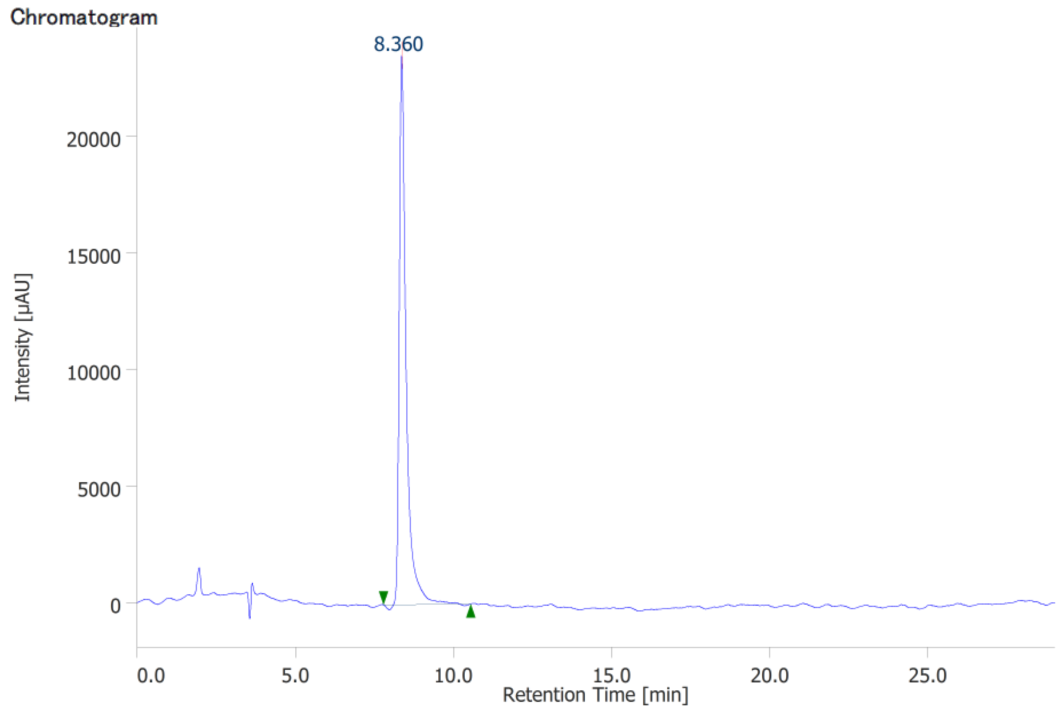
Chromatogram
8.360
Intensity [µAU]
20000
15000
10000
5000
0
0.0
5.0
10.0
15.0
20.0
25.0
Retention Time [min]

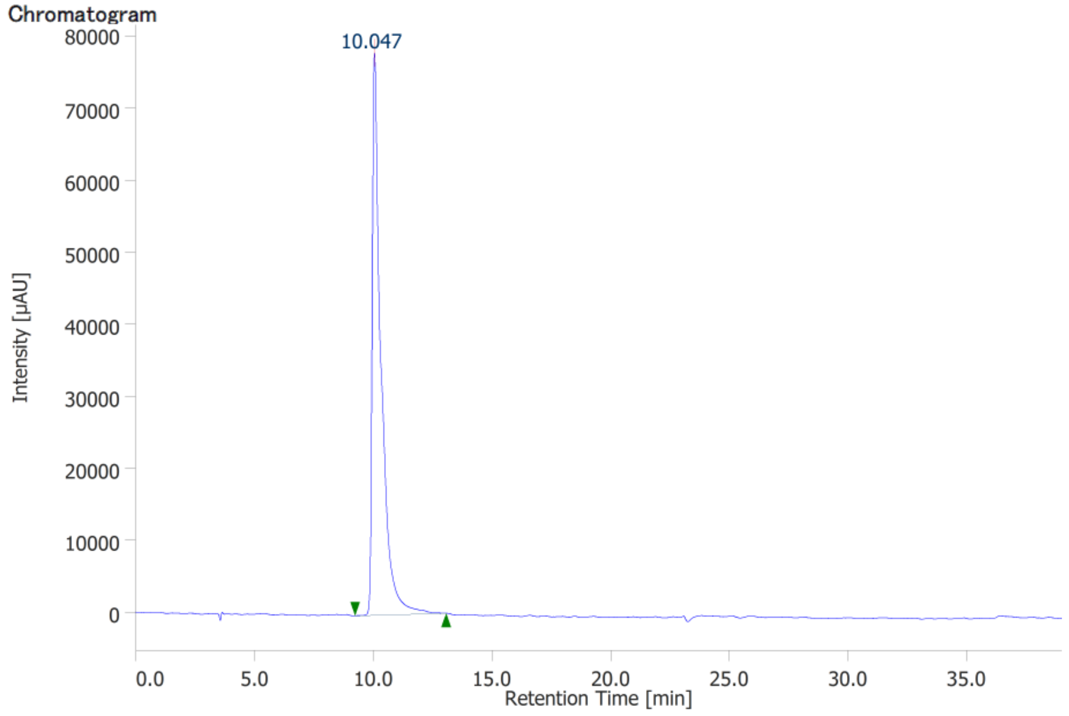
Chromatogram
10.047
Intensity [µAU]
80000
70000
60000
50000
40000
30000
20000
10000
0
0.0
5.0
10.0
15.0
20.0
25.0
30.0
35.0
Retention Time [min]

[(*S*)-**3c** and (*R*)-**3c**]

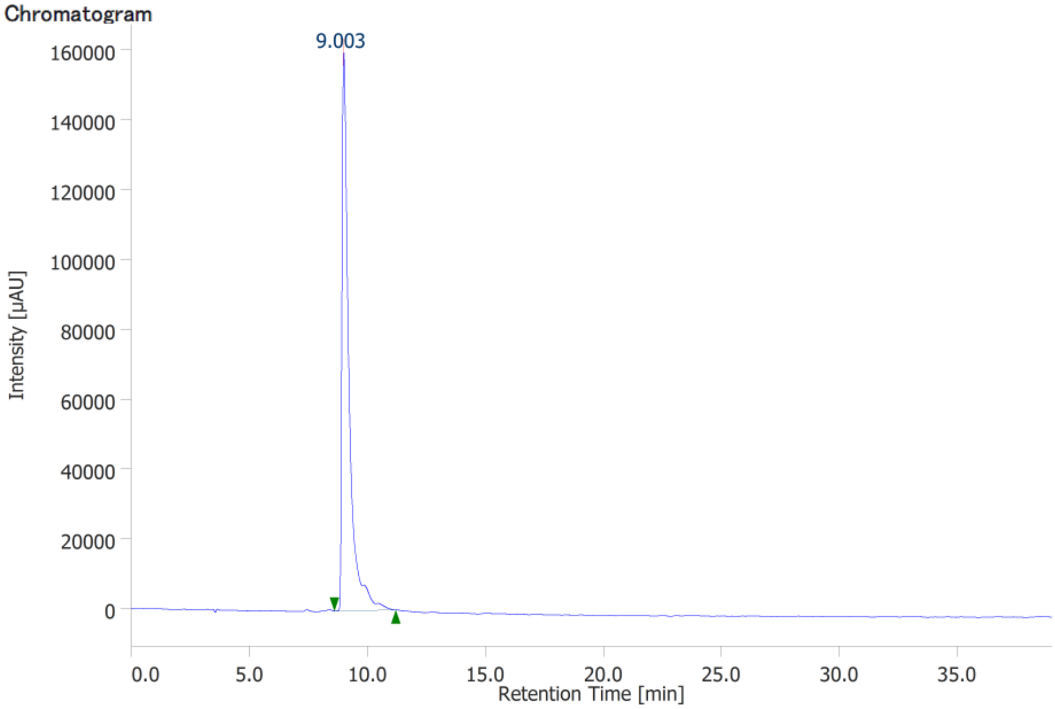
Chromatogram
9.003
160000
140000
120000
100000
80000
60000
40000
20000
0
Intensity [μAU]
0.0
5.0
10.0
15.0
20.0
25.0
30.0
35.0
Retention Time [min]

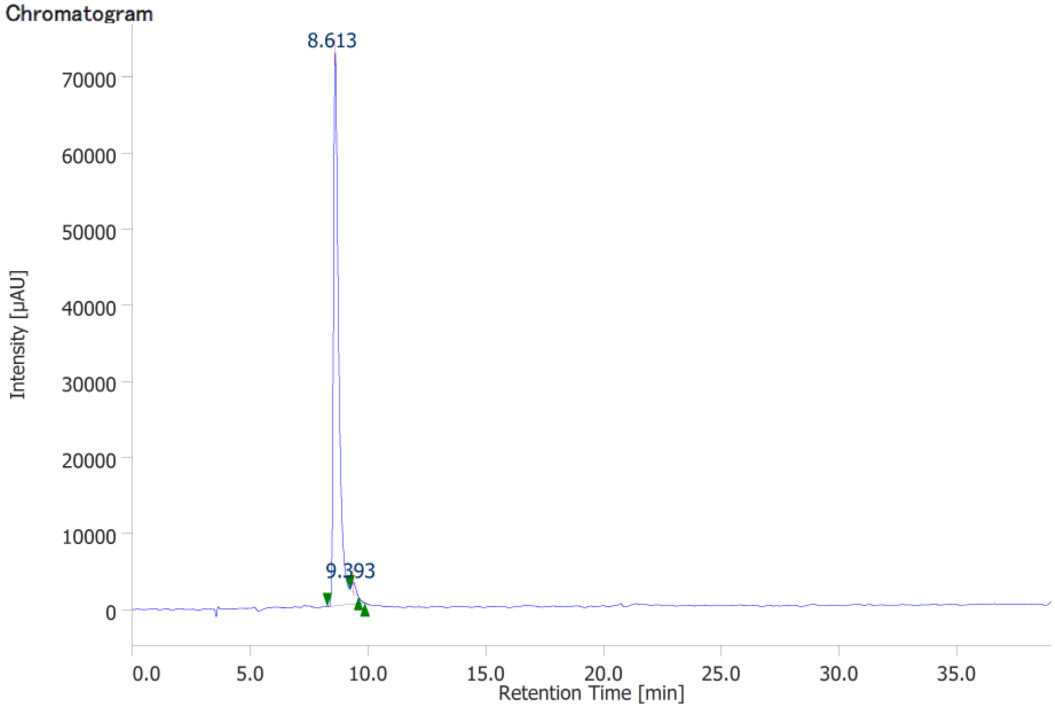
Chromatogram
8.613
9.393
70000
60000
50000
40000
30000
20000
10000
0
Intensity [μAU]
0.0
5.0
10.0
15.0
20.0
25.0
30.0
35.0
Retention Time [min]

[(*S*)-**4a** and (*R*)-**4a**]

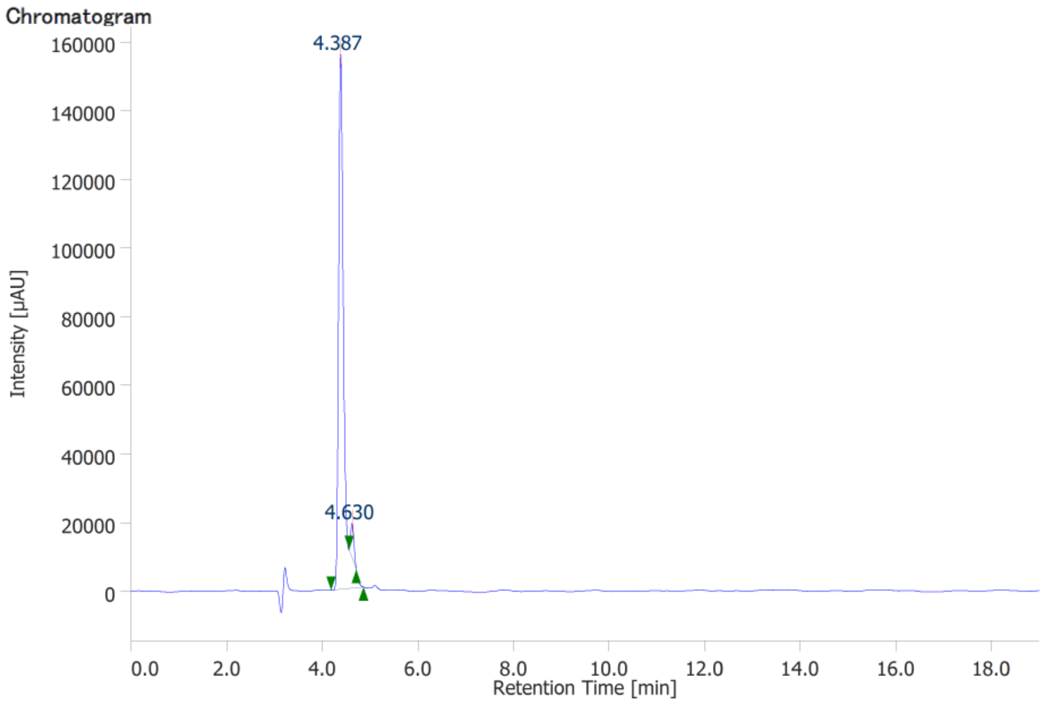
Chromatogram
4.387
4.630
Intensity [µAU]
Retention Time [min]

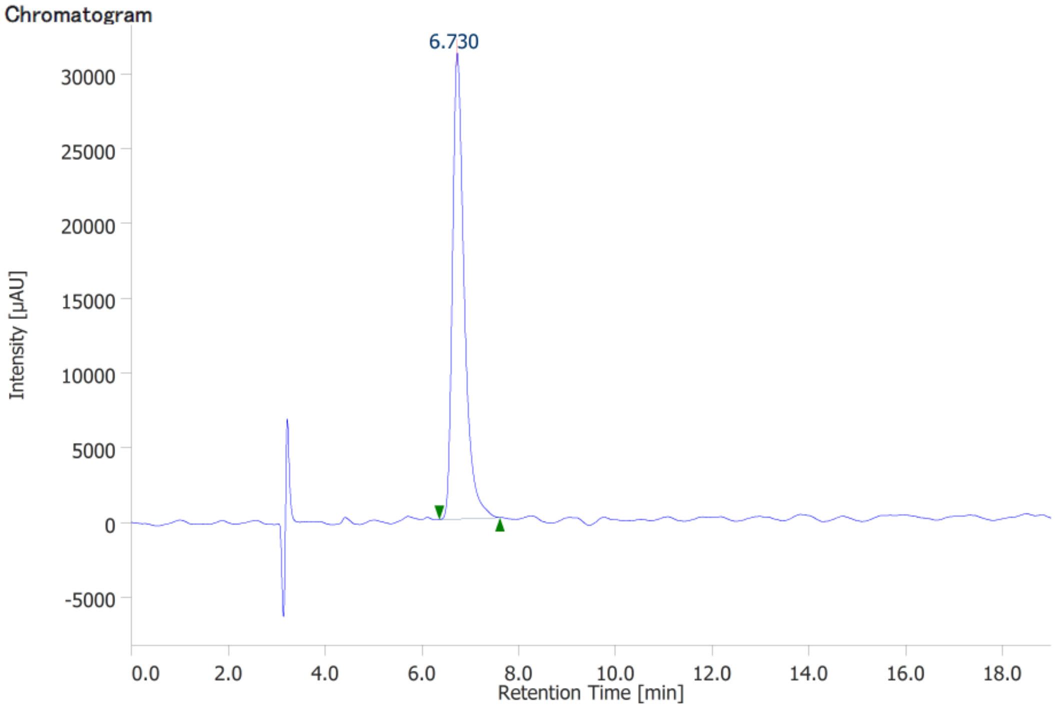
Chromatogram
6.730
Intensity [µAU]
Retention Time [min]

[(*S*)-**4b** and (*R*)-**4b**]

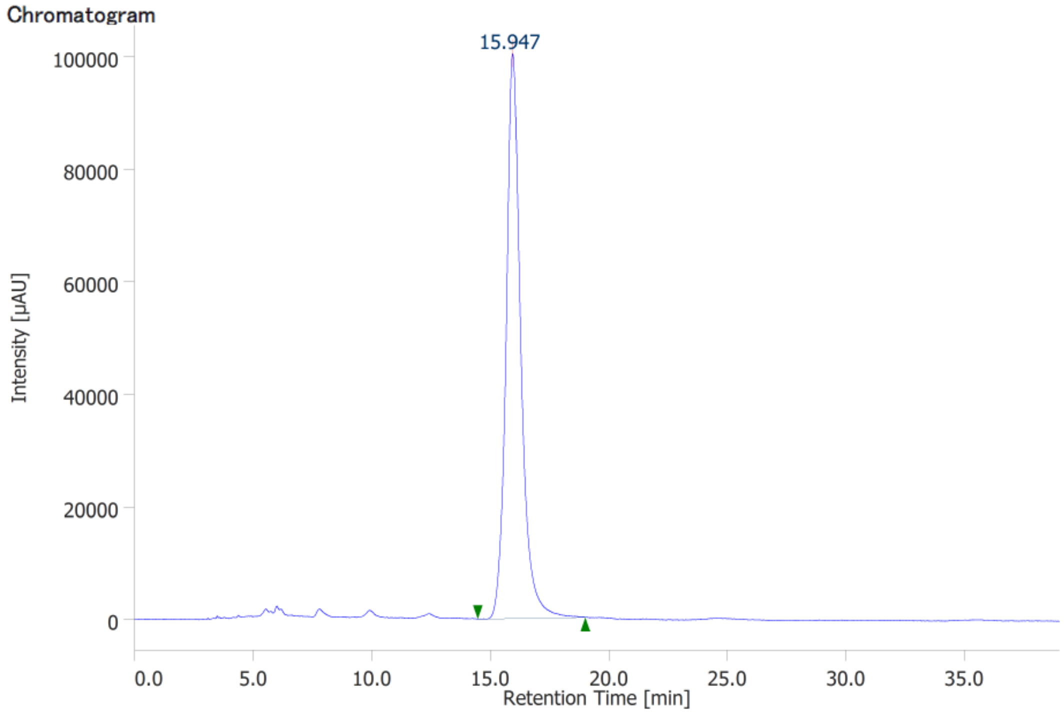
Chromatogram
15.947
Intensity [μAU]
Retention Time [min]

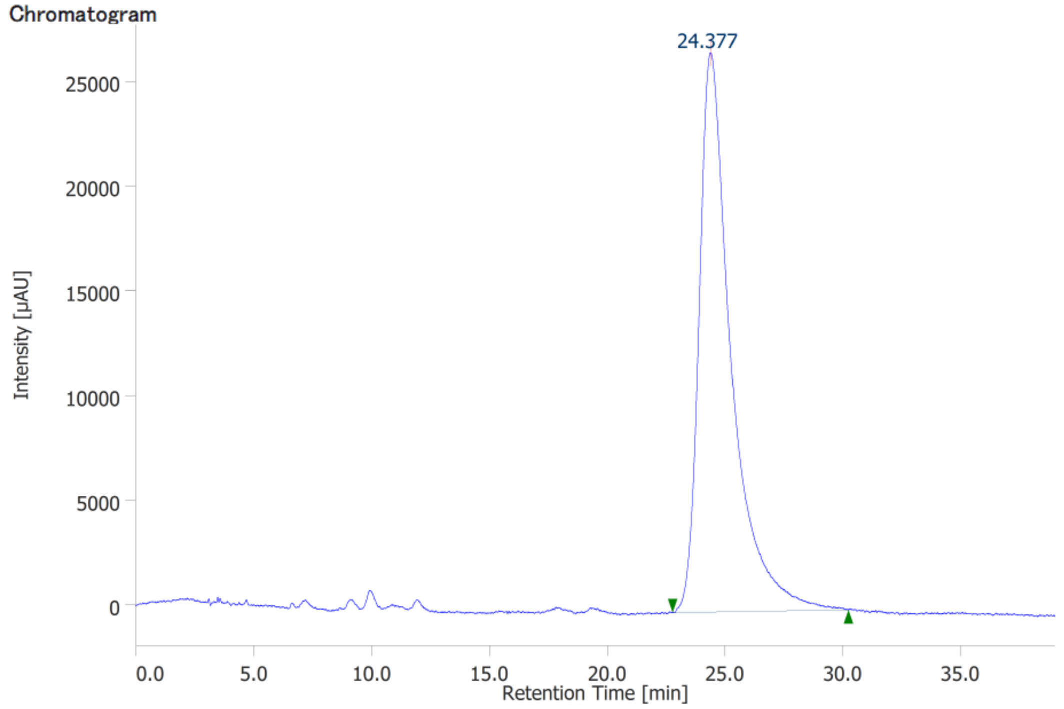
Chromatogram
24.377
Intensity [μAU]
Retention Time [min]

[(*S*)-**4c** and (*R*)-**4c**]

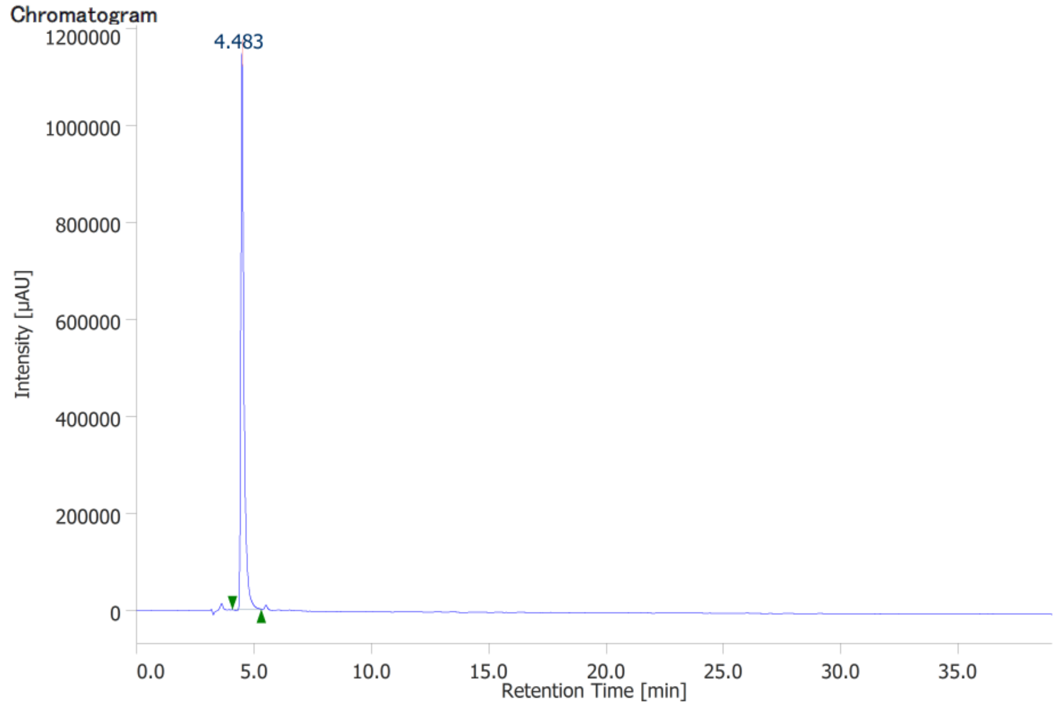
Chromatogram
4.483
1200000
1000000
800000
600000
400000
200000
0
Intensity [μAU]
0.0
5.0
10.0
15.0
20.0
25.0
30.0
35.0
Retention Time [min]

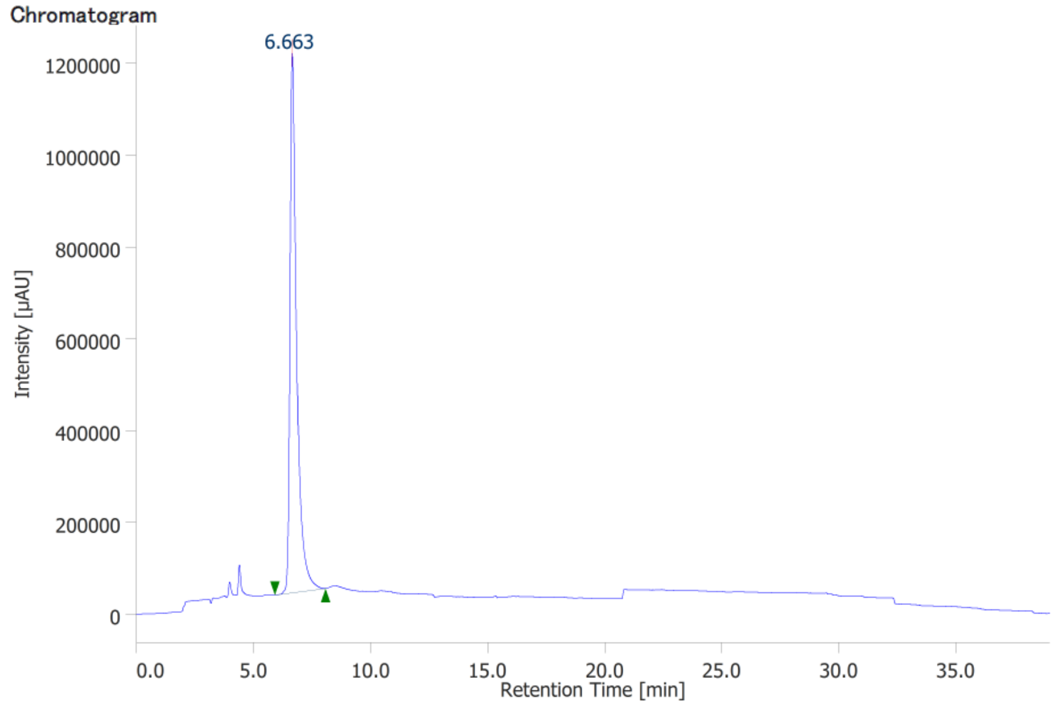
Chromatogram
6.663
1200000
1000000
800000
600000
400000
200000
0
Intensity [μAU]
0.0
5.0
10.0
15.0
20.0
25.0
30.0
35.0
Retention Time [min]

[(*S*)-**5** and (*R*)-**5**]

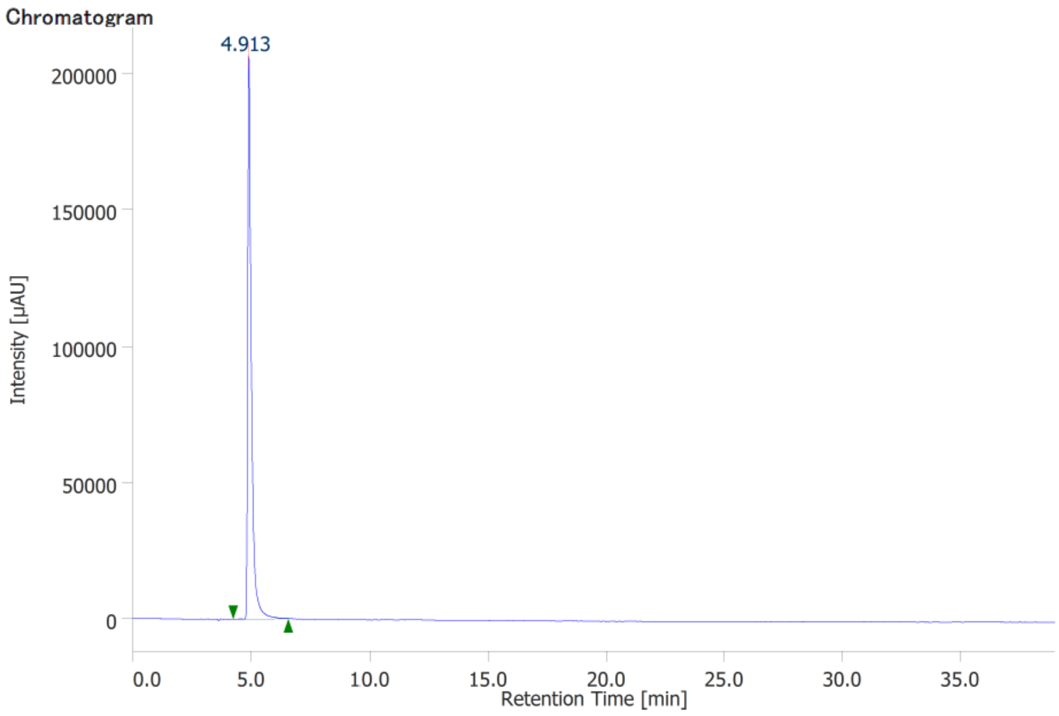

Chromatogram
4.913
200000
150000
100000
50000
0
Intensity [µAU]
0.0
5.0
10.0
15.0
20.0
25.0
30.0
35.0
Retention Time [min]

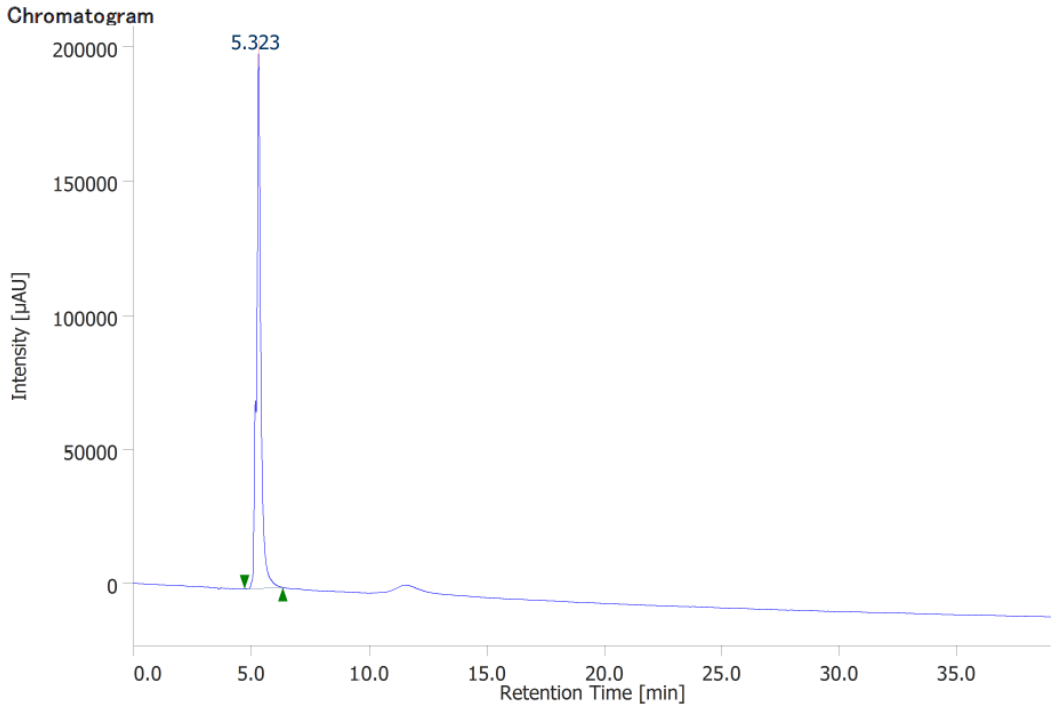

Chromatogram
5.323
200000
150000
100000
50000
0
Intensity [µAU]
0.0
5.0
10.0
15.0
20.0
25.0
30.0
35.0
Retention Time [min]

[(*S*)-**6** and (*R*)-**6**]

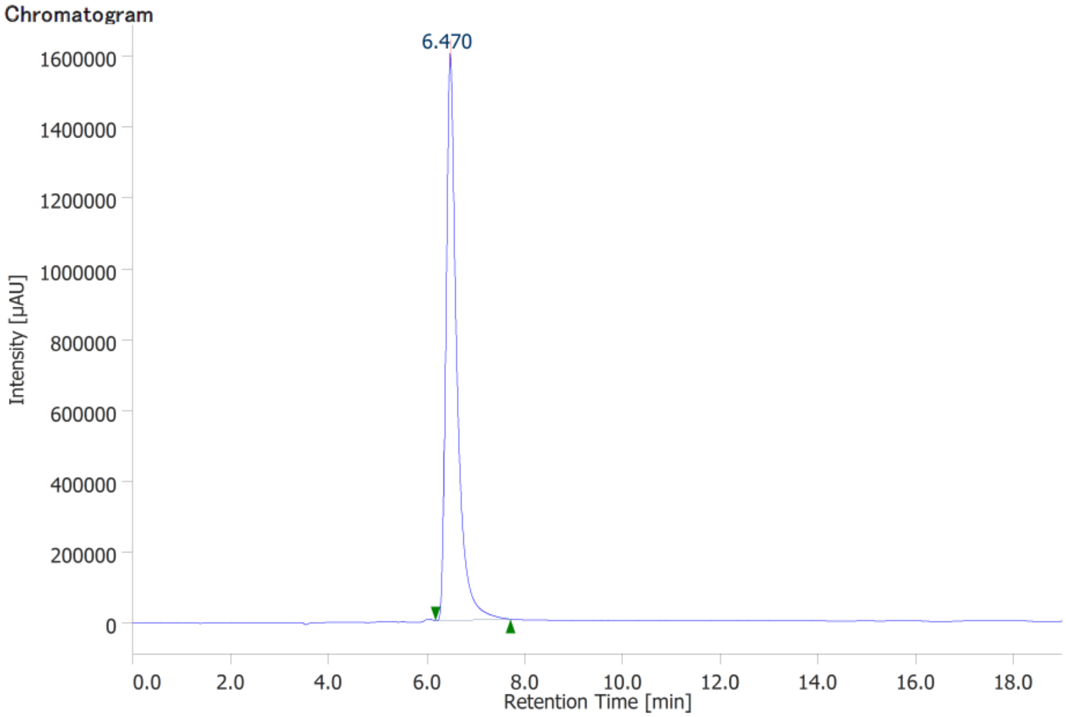

Chromatogram
6.470
1600000
1400000
1200000
1000000
800000
600000
400000
200000
0
Intensity [µAU]
0.0
2.0
4.0
6.0
8.0
10.0
12.0
14.0
16.0
18.0
Retention Time [min]

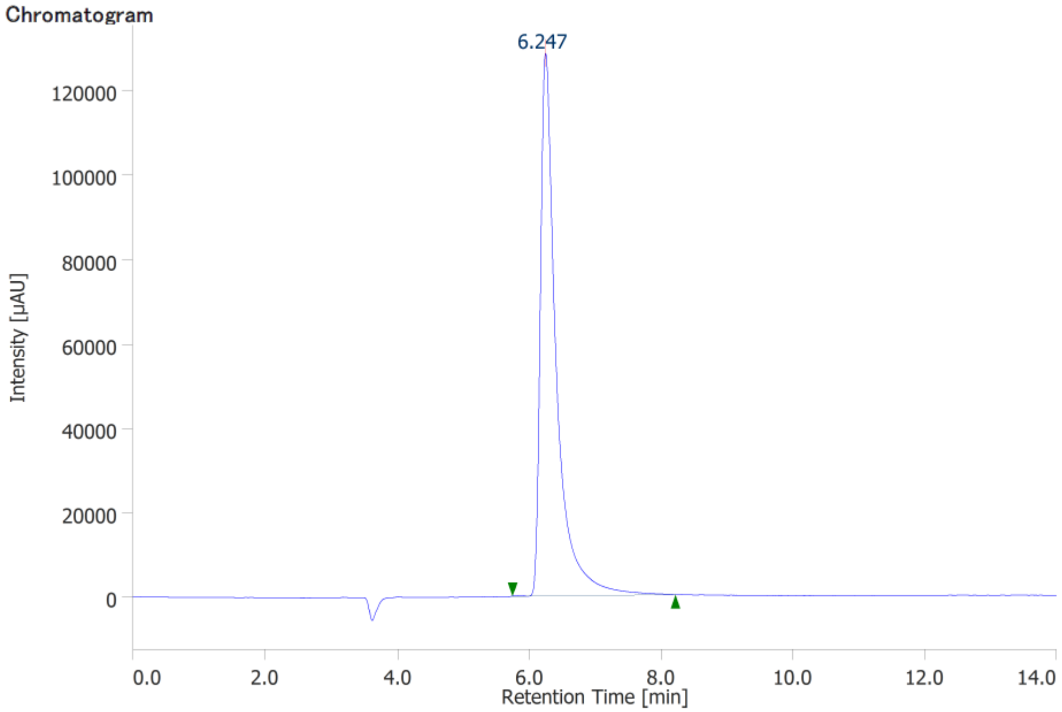

Chromatogram
6.247
120000
100000
80000
60000
40000
20000
0
Intensity [µAU]
0.0
2.0
4.0
6.0
8.0
10.0
12.0
14.0
Retention Time [min]

5. References

[1] Sheldrick, G. M. *Acta Crystallogr. Sect. A,* **2008**, *64*, 112–122.
[2] Farrugia, L. J. J. *Appl. Cryst.,* **2012**, *45*, 849–854.
